# Origins and Molecular Evolution of the NusG Paralog RfaH

**DOI:** 10.1128/mBio.02717-20

**Published:** 2020-10-27

**Authors:** Bing Wang, Vadim M. Gumerov, Ekaterina P. Andrianova, Igor B. Zhulin, Irina Artsimovitch

**Affiliations:** a Department of Microbiology, The Ohio State University, Columbus, Ohio, USA; b The Center for RNA Biology, The Ohio State University, Columbus, Ohio, USA; c Translational Data Analytics Institute, The Ohio State University, Columbus, Ohio, USA; National Cancer Institute

**Keywords:** NusG, RfaH, Spt5, antitermination, transcription

## Abstract

The only universally conserved family of transcription factors comprises housekeeping regulators and their specialized paralogs, represented by well-studied NusG and RfaH. Despite their ubiquity, little information is available on the evolutionary origins, functions, and gene targets of the NusG family members. We built a hidden Markov model profile of RfaH and identified its homologs in sequenced genomes. While NusG is widespread among bacterial phyla and coresides with genes encoding RNA polymerase and ribosome in all except extremely reduced genomes, RfaH is mostly limited to *Proteobacteria* and lacks common gene neighbors. RfaH activates only a few xenogeneic operons that are otherwise silenced by NusG and Rho. Phylogenetic reconstructions reveal extensive duplications and horizontal transfer of *rfaH* genes, including those borne by plasmids, and the molecular evolution pathway of RfaH, from “early” exclusion of the Rho terminator and tightened RNA polymerase binding to “late” interactions with the *ops* DNA element and autoinhibition, which together define the RfaH regulon. Remarkably, NusG is not only ubiquitous in *Bacteria* but also common in plants, where it likely modulates the transcription of plastid genes.

## Abstract (precis)

In all domains of life, NusG-like proteins make contacts similar to those of RNA polymerase and promote pause-free transcription yet may play different roles, defined by their divergent interactions with nucleic acids and accessory proteins, in the same cell. This duality is illustrated by Escherichia coli NusG and RfaH, which silence and activate xenogenes, respectively. We combined sequence analysis and recent functional and structural insights to envision the evolutionary transformation of NusG, a core regulator that we show is present in all cells using bacterial RNA polymerase, into a virulence factor, RfaH. Our results suggest a stepwise conversion of a NusG duplicate copy into a sequence-specific regulator which excludes NusG from its targets but does not compromise the regulation of housekeeping genes. We find that gene duplication and lateral transfer give rise to a surprising diversity within the only ubiquitous family of transcription factors.

## INTRODUCTION

**FIG 1:**
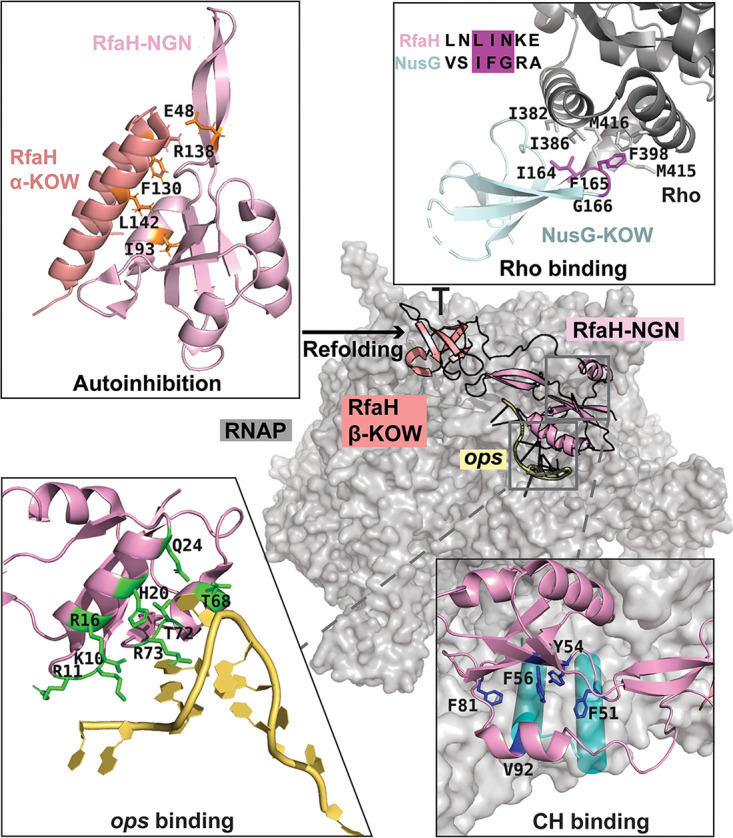
RfaH and NusG interactions with the transcription machinery. Autoinhibited RfaH interacts with the *ops* DNA hairpin formed on the RNAP surface, transforms into an active NusG-like state, and binds to the β′ clamp helices (CHs); NusG makes similar but weaker contacts with RNAP (see Fig. S1 in the supplemental material). The NusG-KOW domain binds to Rho and promotes termination. Residues that make important functionally validated contacts are shown as sticks. PDB accession numbers are as follows: NusG-Rho binary complex, 6DUQ; autoinhibited RfaH, 5OND; RfaH bound to *ops*-paused transcription elongation complex, 6C6S.

RNA synthesis by RNA polymerase (RNAP) must be elaborately controlled in response to diverse intracellular and environmental cues. However, cellular RNAPs bind to DNA largely nonspecifically and depend on numerous accessory proteins to determine when and where to start, pause, and stop RNA synthesis. Among hundreds of transcription factor families, only NusG-like regulators are present in all domains of life (1). These proteins have similar structural cores (Fig. 1) consisting of a NusG N-terminal (NGN) domain and a C-terminal domain with a Kyprides-Ouzounis-Woese (KOW) motif (2); eukaryotic Spt5 proteins have several KOW domains and additional regulatory regions (3). Consistently with their common evolutionary origin and function, NGNs of NusG homologs from archaea, bacteria, and eukaryotes bind to the same sites on the elongating RNAP (4–6), composed of the clamp helix (CH) domain in the largest RNAP subunit (β′ in *Bacteria*) and the gate loop in the second-largest subunit (β in *Bacteria*). Once bound, NusG proteins (or their NGNs alone) promote processive, pause-free RNA synthesis (7), a function thought to be particularly important for the synthesis of very long RNAs. Recent structural studies revealed a common molecular basis for antipausing activity among all NusG-like proteins (4, 5, 8).

10.1128/mBio.02717-20.1 FIG S1 Structural comparison of NusG- and Spt5-NGN. (A) Structure of E. coli NusG-RNAP complex (PDB accession no. 6C6U). The β′ clamp helix (CH)-binding residues are indicated in blue. The position of the β-hairpin loop was determined by superposition over the NusG structure (PDB accession no. 2K06). (B) Structure of Pyrococcus furiosus Spt5-NGN-RpoE′′-RNAP complex (PDB accession no. 3QQC). RPB1 CH-binding residues are shown in blue. RpoE′′-binding residues are in magenta, with the invariant E49 involved in the acid-dipole interaction with Spt4 shown in yellow. (C) Alignment of E. coli NusG-NGN (NCBI accession no. NP_418409.1) and P. furiosus Spt5-NGN (WP_011013134.1). NusG (top) and Spt5 (bottom) logos were generated from sequences collected from GTDB_reps (sequences were reduced at 90% identity by CD-hit (88); a total of 4,220 NusG and 154 Spt5 sequences were included in the final alignment). CH-binding residues are indicated with blue circles; E49 is indicated with a yellow circle. The β hairpin loop and RpoE′′-binding region are shown in green and magenta boxes, respectively. Download FIG S1, PDF file, 0.9 MB. Copyright © 2020 Wang et al. 2020 Wang et al. This content is distributed under the terms of the Creative Commons Attribution 4.0 International license.

NusG homologs comprise two distinct families, which are correlated with the architecture of their respective target RNAPs (Fig. S1). In bacteria, NusG binds to a “minimal” RNAP typically composed of five subunits and promotes uninterrupted RNA synthesis (9). Although NusG can interact with other proteins as part of specialized antitermination complexes (10), it does not require any accessory factors for binding to RNAP. In contrast, in eukaryotes and archaea, which have more complex 12+ subunit RNAPs, Spt5 has an obligatory partner, a small zinc finger protein, Spt4 (called RpoE in archaea). Spt4 and Spt5 form an extensive interface with several conserved residues (11, 12); among them, a universally conserved Glu residue is essential for Spt4/5 binding, and its replacement of Gln (the corresponding residue in NusG) abolishes their interactions (13). Together, Spt4/5 (DSIF in metazoans) promote transcription elongation similarly to NusG (8, 14). Spt4 was long thought to simply buttress Spt5 stability (11, 14), but recent structural data suggest that it also contributes to maintaining RNAP processivity, for example, during transcription through nucleosomes (15). Spt4 binds to Spt5-NGN opposite the RNAP interaction surface, and several conserved basic residues in Spt4 form a part of the upstream DNA channel (4). In NusG, a positively charged β-hairpin loop is positioned similarly to Spt4 (5, 16) and may interact with the upstream DNA duplex (17); large modulatory domains present in place of the β-hairpin in some NusG proteins may contribute to DNA interactions (2, 13). The presence of the β-hairpin is incompatible with an auxiliary protein binding to NusG in a manner similar to the way it binds to Spt4 (11); accordingly, Spt5 proteins do not have insertions at this position (Fig. S1). Within a given cell, NusG and its paralogs can be viewed as alternative transcription elongation factors which compete for binding to RNAP, similarly to σ initiation factors (7). This analogy is strengthened by the fact that NusG and σ (or Spt5 and TFE in *Archaea*) share the binding site on RNAP (18, 19). However, in stark contrast to σ factors, which perform the same function at their cognate promoters, NusG-like proteins play surprisingly multifaceted roles, as can be illustrated in Escherichia coli, which encodes two of the best-characterized members of this family: an abundant and essential housekeeping NusG protein and its scarce nonessential specialized paralog RfaH (20). NusG promotes productive RNA synthesis as part of antitermination complexes (10) or by coupling transcription to translation via direct contacts with the ribosome (21, 22). Yet if RNA is useless or potentially harmful, as is the case with many xenogenes, the NusG-KOW domain interacts with the termination factor Rho to induce its early release from RNAP (23); in fact, silencing of xenogenes constitutes an essential function of E. coli NusG (24). RfaH plays an opposite role; it activates expression of xenogenes (7), many of which encode virulence factors, and is required for virulence in enteric pathogens (7).

While NusG associates with RNAP transcribing all operons (20), RfaH is recruited to its targets only at operon polarity suppressor (*ops*) elements in the nontemplate DNA strand in the transcription bubble (20, 25). The *ops* signal halts RNAP to provide more time for RfaH recruitment and forms a short DNA hairpin that interacts with the RfaH-NGN to induce RfaH transformation from an autoinhibited state to an activated state (26) (Fig. 1). Once bound, RfaH excludes NusG from the transcribing RNAP, thereby insulating it from Rho, and activates translation by recruiting the ribosome (20, 27). Extensive genetic, biochemical, and structural data available for RfaH and NusG provide a detailed molecular context for understanding their effects on gene expression. While both proteins interact with similar regions on RNAP, RfaH binds much more tightly (5), giving RfaH advantage to compete with 100-fold more abundant NusG (28), and only NusG interacts with Rho (23). These proteins make similar contacts with the ribosomal protein S10 (21, 27), but in the case of RfaH, a dramatic metamorphosis (in which the entire RfaH-KOW motif refolds from an α-helical hairpin observed in free, autoinhibited RfaH [29] to a β-barrel) is required to expose the residues that interact with S10 (27). This switch is triggered when RfaH binds to the *ops*-paused RNAP (30).

In contrast, relatively little is known about NusG homologs present in diverse bacteria (31). An emerging view is that specialized NusG paralogs (NusG^SP^s) function as dedicated antiterminators of long, difficult-to-express gene clusters required for adaptation to diverse environments, including human hosts. Bacterial genes shown to be dependent on NusG^SP^s for expression encode adhesins, capsular polysaccharides, conjugation machinery, polyketide antibiotics, and toxins (7). While RfaH is recruited to *ops* sites in the leader regions of several unlinked chromosomal targets (20), some NusG^SP^s are encoded within the operons that they regulate (32, 33) and their modes of recruitment are unknown.

In this work, we set out to reconstruct the origins and evolutionary history of RfaH and its relationship to NusG, expanding previous phylogenetic analysis (31) to incorporate the growing number of sequences in public databases and recent experimental insights into the functions of these proteins. Using sensitive profile searches, including those with a newly constructed profile model for RfaH, we revealed the phyletic distribution of NusG and RfaH across the tree of life. Our results show that ancient and recent gene duplication, horizontal gene transfer, and rapid functional divergence of paralogs underlie the evolution of the NusG family. One of these NusG duplications, which occurred in *Proteobacteria*, led to the emergence of RfaH. Changes within the key functional regions of NusG paralogs suggest that nascent NusG duplicates have gradually morphed into fully specialized RfaH-like regulators by losing contacts with Rho first and acquiring sequence-specific DNA contacts last. We found that NusG homologs are encoded in most plants and photosynthetic protists and in all except severely reduced bacterial genomes. These results support a notion that NusG modulates transcription in nearly every cell that utilizes RNAP of the bacterial type.

## RESULTS AND DISCUSSION

In addition to housekeeping NusG/Spt5 proteins, their specialized paralogs are known in bacteria and eukaryotes (31, 34). These paralogs are assumed to have arisen by gene duplication, followed by adaptation to unique regulatory demands, e.g., upregulation of virulence genes during bacterial pathogenesis, a key function of several NusG paralogs in Gram-negative bacteria. Among many bacterial NusG paralogs (31), only a handful have been characterized, but even cursory analyses revealed a surprising diversity in their primary sequence, function, and even structure. NusG-like proteins modulate gene expression through a network of contacts with RNAP, nucleic acid signals, and ribosome (7). In-depth studies of E. coli NusG and RfaH provided atomic-level details of these interactions and identified dramatic conformational changes that underlie their differential recruitment mechanisms (Fig. 1).

### New RfaH model.

**FIG 2:**
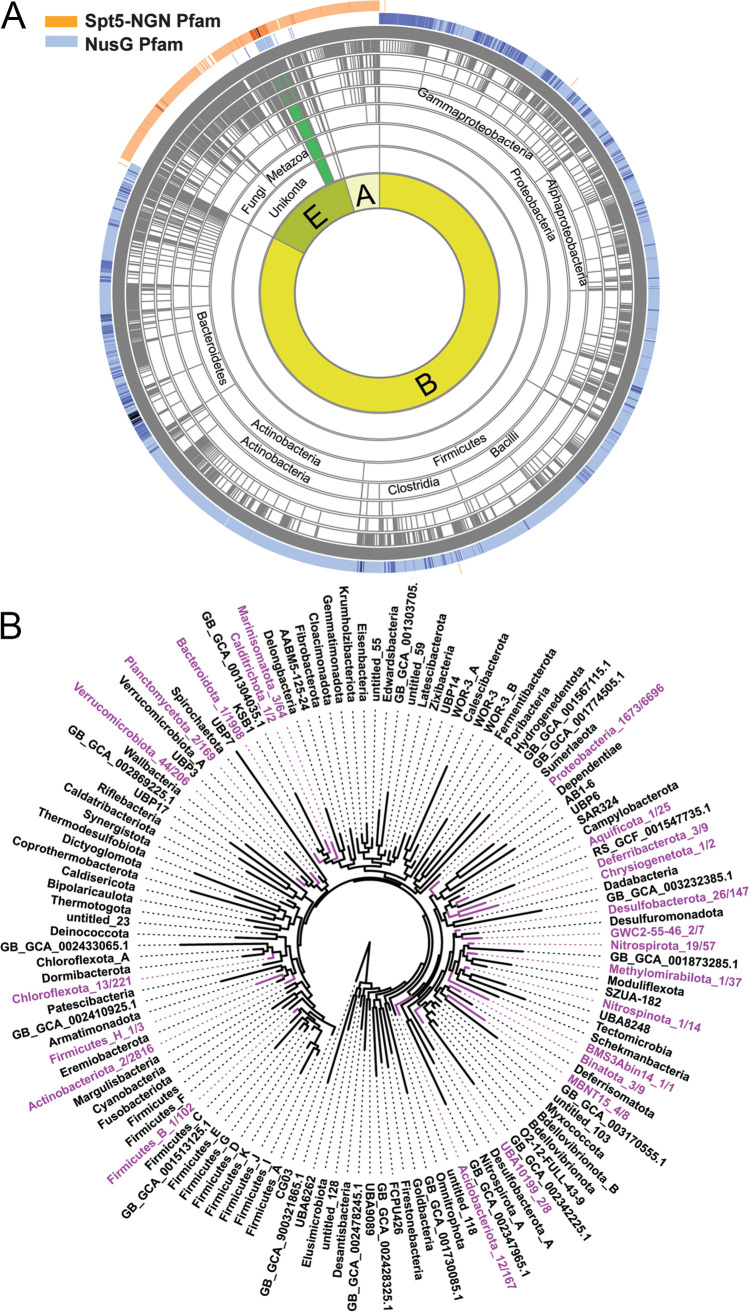

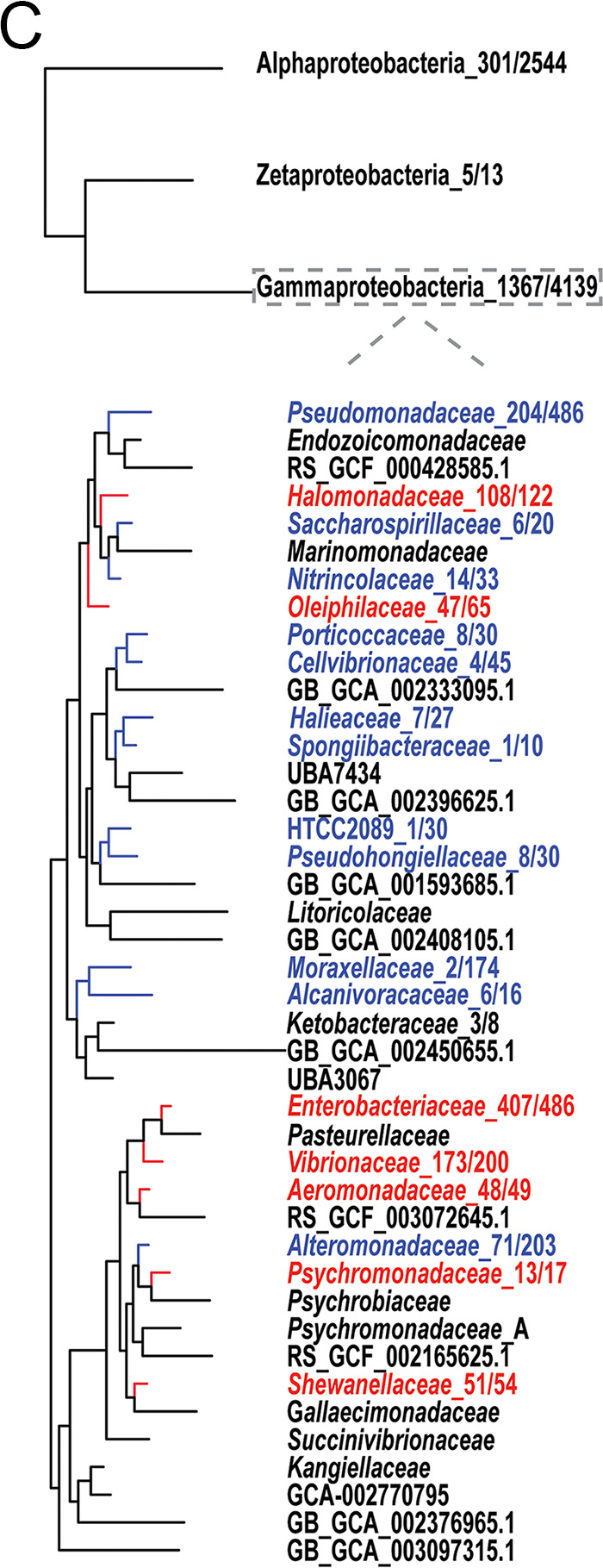
The distribution of NusG-like factors. (A) NusG/Spt5 factors were identified using NusG and Spt5-NGN Pfam models, respectively, in Aquerium (93; http://aquerium.zhulinlab.org/). The outer ring shows the number of hits; the darker the color, the more hits it represents. The inner rings represent the major taxonomic ranks and supergroups for eukaryotes (93). E, *Eukaryota*; A, *Archaea*; B, *Bacteria*. Plantae are green. (B) RfaH distribution in bacteria on the phylum level. The genome tree was downloaded from AnnoTree (77; http://annotree.uwaterloo.ca/). Phyla with representatives that contain RfaH (based on hits with our new model) are highlighted in purple. Numbers appended after taxons indicate the number of genome hits divided by the total number of genomes. (C) RfaH distribution in *Proteobacteria*. The percentages of genome hits were calculated for RfaH-containing families with ≥10 genomes. Families with >50% hits are shown in red, and those with <50% hits are shown in blue. A genome tree of representative *Gammaproteobacteria* is shown. This and other genome trees are maximum-likelihood trees inferred from the alignment of 120 ubiquitous single-copy proteins (53).

NusG homologs are widely distributed across all three domains of life (Fig. 2A), but they are very diverse, likely reflecting adaptation to very different niches. This diversity necessitates the use of robust models to investigate the evolution of the NusG family. We needed a model that can reliably distinguish RfaH proteins from the rest of the NusG family. Pfam (35), the leading protein domain database, does not have a specific RfaH model, and its NusG model (PF02357) cannot distinguish NusG from its paralogs. An RfaH-specific model is available in TIGRfam, but this model (TIGR01955) was constructed using only five sequences and was last modified in 2011. Using Pfam guidelines, we built a new hidden Markov model (HMM) profile for RfaH based on 260 seed sequences (see Methods in Fig. S2 in the supplemental material). The new RfaH model detected 4,173 sequences in the UniProtKB database (36), while the TIGRfam RfaH model detected only 2,955. The new RfaH model has been deposited in the MiST database (37) and will be available in its next release.

10.1128/mBio.02717-20.2 FIG S2 Example of reciprocal best BLAST hits. Protein sequences were used as queries in BLAST against each other’s genome. If two sequences find each other as the best-scoring match in each other’s genome, they will be called reciprocal best BLAST hits and connected with a line. The number of mutual connections is indicated inside circles. Black circles, chromosomal RfaH; purple circles, plasmid RfaH. RfaH is presented as a genus name and protein NCBI accession number. A high number of mutual connections was observed for every representative, indicating that these representatives belong to the same orthologous groups. Download FIG S2, PDF file, 0.7 MB. Copyright © 2020 Wang et al. 2020 Wang et al. This content is distributed under the terms of the Creative Commons Attribution 4.0 International license.

### Distribution of housekeeping NusG.

Although presumed to be ubiquitous, NusG was absent in a few (7 out of 711) representatives of COG0250 (38; https://www.ncbi.nlm.nih.gov/COG/). We extended this analysis to a data set of nearly 20,000 representative bacterial and archaeal genomes from the Genome Taxonomy Database (39), to which we refer here as GTDB_reps (see Materials and Methods). In *Archaea*, Spt5 is widespread (Fig. 2A) but not ubiquitous: using Spt5-NGN as a model, we identified Spt5 in only 789 out of 847 archaeal genomes. A similar trend was observed in bacteria, where 6% of bacterial GTDB_reps genomes had no identifiable NusG proteins (Data Set S1A and -B). The lack of NusG/Spt5 may be due to (i) incomplete genome assemblies or sequencing errors, (ii) gene loss, or (iii) the low sensitivity of the search model. To evaluate these scenarios, we analyzed NusG homolog distribution in ∼130,000 bacterial genomes from the NCBI nonredundant database. Among them, 1,879 appeared to lack NusG homologs (Data Set S1C), but no clear pattern has emerged. Moreover, approximately the same fraction of genomes lacked SecE, RecA, and essential ribosomal proteins L5, L6, S2, and S7 (Data Set S1C). The absence of essential core genes in a significant fraction of genomes is most likely due to technical issues arising during genome sequencing/assembly and exposes limitations of this broad-stroke approach, necessitating in-depth analysis. By analyzing 13,140 NusGs (Data Set S1A) using TREND (40; http://trend.zhulinlab.org), we found that *nusG* is invariably present within a highly conserved operon that encodes the protein translocase SecE and 50S ribosomal proteins. Thus, we further investigated *secE-nusG?-rplK-rplA* genomic loci in 183 genomes that appear to lack NusG but contain SecE and ribosomal protein L1 (*rplA*), as well as RecA and L5, L6, S2, and S7 (Data Set S1D).

**FIG 3:**
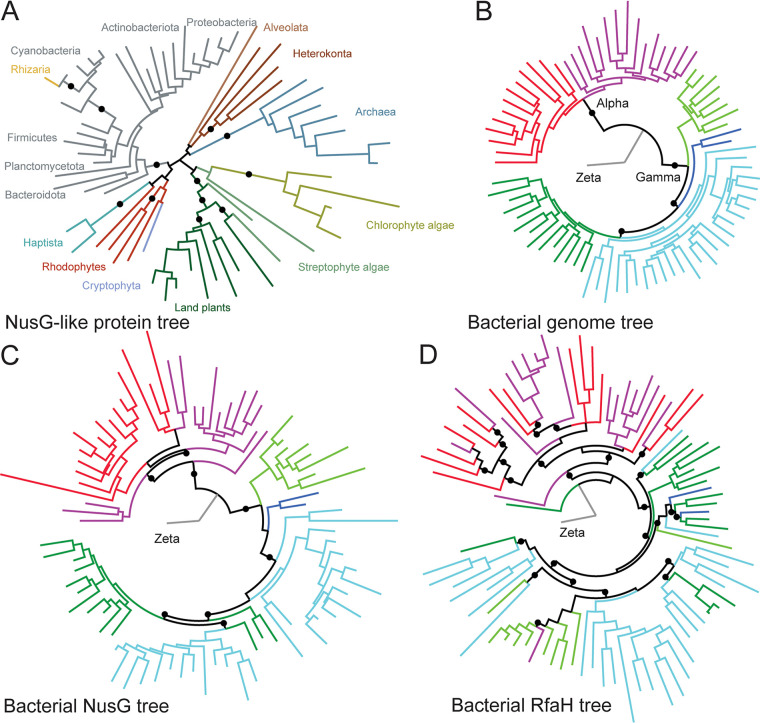
Maximum-likelihood phylogenetic trees. (A) NusG-like proteins are widespread. (B to D) Topology of bacterial trees, with monophyletic groups colored in the genome tree (B). The two clades of *Alphaproteobacteria* (Alpha) are red and purple; one clade of *Zetaproteobacteria* (Zeta) is gray. The remaining clades belong to *Gammaproteobacteria* (Gamma). The branches of NusG (C) and RfaH (D) trees are colored according to the genome tree. Black dots indicate bootstrap values of >50% (A) or >70% (B to D).

10.1128/mBio.02717-20.10 DATA SET S1 Data sets generated in this study. (A) NusG collected by the NusG TIGRfam model in GTDB_reps. (B) Statistics of genome hits of NusG TIGRfam in GTDB_reps. (C) NusG homolog distribution in 129,663 bacterial genomes. In model hits, 0 indicates no hits and 1 indicates hits. The total genome hits of different models are presented. (D) Genomes that lack NusG Pfam hits but have other core selected proteins. The NusG family absence was manually checked for complete genomes (those have a Complete Genome assembly level). (E) NusG distribution in bacterial endosymbionts. The absence of NusA/NusG/RpoZ (ω) is highlighted in yellow; in contrast, some core genes, such as those encoding an essential GroELS chaperone system, are present in all genomes. One representative of each species is shown except for “*Candidatus* Sulcia muelleri,” whose patterns differ among 39 sequenced representatives. Only a small number of genomes larger than 0.5 Mb are shown. (F) NusG homolog detection in Plantae and Chromista. The presence of a chloroplast transit signal (CTS) was predicted by the ChloroP 1.1 server (http://www.cbs.dtu.dk/services/ChloroP/). Sequences with a CTS are highlighted in green. (G) Comparative genome analysis of Plantae and Chromista NusG homologs. (H) RfaHs collected by the new RfaH model in GTDB_reps. (I) Statistics of genome hits of a new RfaH model in GTDB_reps. (J) Representatives for topology analysis (Fig. 3B to D). (K) RfaH distribution in subspecies of *Pseudomonadaceae*. (L) Representatives used for building the phylogenetic trees of UpxY, RfaH, and NusG (Fig. S7). (M) Representatives of the molecular evolution study (Fig. 5). (N) The eight RfaH clusters (CLs) generated by Markov clustering. Download Data Set S1, XLSX file, 6.4 MB. Copyright © 2020 Wang et al. 2020 Wang et al. This content is distributed under the terms of the Creative Commons Attribution 4.0 International license.

To ensure genome completeness, we selected only those NusG-less representatives that have a “complete genome” assembly level (12 total). Analysis of the *secE-nusG?-rplK-rplA* operons identified 1-nt frameshifts in the *nusG* open reading frames (ORFs) in 11 genomes. Among these, 9 have sequences of the same species in which *nusG* is intact, whereas two genomes are present in single copies, albeit with sequences of their NusG-encoding close relatives available (Data Set S1D). The *nusG* gene was deleted from “*Candidatus* Evansia muelleri,” an endosymbiont with a severely reduced 0.36-Mbp genome. Consistently, six out of seven NusG-less COG0250 representatives have genomes smaller than 0.28 Mbp, whereas the remaining genome is incomplete.

These findings suggest that reduced genome endosymbionts may function with reduced transcription machinery. In E. coli, a transcribing five-subunit core RNAP (α_2_ββ′ω) associates with NusA and NusG across the entire genome (20); both Nus factors are essential in wild-type E. coli. We wondered if NusA and ω, which acts as a chaperone and is not essential (41), could also be absent in endosymbionts. We analyzed complete genomes ranging from 0.11 to 5+ Mbp (Data Set S1E). We found that all genomes smaller than 0.2 Mbp did not encode NusG or NusA, whereas genomes larger than 0.36 Mbp encoded both proteins. In genomes bridging these groups, all possible NusA/NusG distribution patterns were observed, sometimes varying between genomes of the same species. Interestingly, ω is absent from many endosymbionts (Data Set S1E), as well as from some free-living bacteria (COG1758). We conclude that all bacterial genomes with the exception of severely reduced genomes encode NusA and at least one NusG family protein. While this conclusion may appear trivial in the case of the “ubiquitous” regulator, *nusG* has been shown to be dispensable in some model organisms grown under laboratory conditions, such as Bacillus subtilis (42), and can even be deleted in E. coli lacking toxic prophages (43), albeit at a marked fitness cost. Clearly, bacterial survival and adaptation to complex environmental conditions impose requirements different than those of growth in rich medium at an optimal temperature.

### Expansion of NusG taxonomic presence.

Realizing that NusG is not restricted to prokaryotes (Fig. 2A), we investigated its distribution further. Using phylogenetic profiling with the most recent Archaeplastida taxonomy (44), we established that, in addition to Spt5, NusG homologs are encoded in the genomes of all major land plant and algal lineages except for some green algal species (Data Set S1F). In addition to identifying NusG homologs in Archaeplastida, we identified them in the genomes of various phyla of photosynthetic chromists (Fig. 3A and Data Set S1F). All genomes in which we could not identify NusG were of poor quality and only partial. All identified NusG homologs in Plantae and Chromista are encoded in the nuclear genomes, except with the *Paulinella* genus. We hypothesize that these “bacterial” regulators have been retained to assist RNA synthesis by plastid-encoded RNA polymerase (PEP) of the bacterial type. Several lines of evidence support this hypothesis. First, a NusG homolog of a model organism, Arabidopsis thaliana, annotated as “plastid transcriptionally active 13” protein (pTAC13), has been identified as a component of the active transcriptional machinery in chloroplasts (45). Second, a Rho ortholog has been shown to terminate transcription by *Arabidopsis* PEP (46). Finally, ChloroP 1.1 (47) predicted the presence of a chloroplast transit signal in several newly identified NusG-like proteins (Data Set S1F). Pervasive plastid transcription has been documented in protists (48, 49).

In rhizarian amoebas of the *Paulinella* genus, *nusG* is carried in the remnants of a bacterial genome: a photosynthetic organelle called chromatophore. *Paulinella* representatives formed an evolutionarily recent symbiotic relationship with a photosynthetic cyanobacterium independently from the primary endosymbiosis that gave rise to plastids in Archaeplastida (50, 51). Our phylogenetic analyses revealed that *Paulinella* NusG is nested within the bacterial NusG cluster in the branch with *Synechococcus* (Fig. 3A), which is considered to be the ancestor of chromatophores (52).

Phylogenetic analysis showed that eukaryotic NusG sequences from Plantae and Chromista formed clusters separate from bacterial and archaeal NusGs (Fig. 3A). Comparative genome analysis using plant and Chromista NusG proteins did not identify any single bacterial group to which all eukaryotic NusG proteins would be most similar (Data Set S1G). These data strongly suggest the presence of a progenitor NusG-like protein in the last universal common ancestor (LUCA).

### RfaH evolution events.

A total of 1,922 RfaH proteins were found in 23 out of 117 phyla of *Bacteria* (Fig. 2B; Data Set S1H and -I), with ∼95% of RfaHs being found in *Proteobacteria*. Seventy percent and 18% of *rfaH* genes are found in *Gammaproteobacteria* and *Alphaproteobacteria*, respectively (Fig. 2C; Fig. S3). Further analysis revealed that families with a high percentage of hits for RfaH are clustered around the *Enterobacteriaceae* (Fig. 2C; Fig. S4). Although in the majority of lineages, the *rfaH* gene is likely a result of vertical evolution, the presence of *rfaH*-like genes on plasmids and prophages suggests that some RfaHs were acquired via horizontal gene transfer (HGT). To evaluate this possibility, we compared the topologies of phylogenetic trees (Fig. 3B to D; Data Set S1J). The three classes of *Proteobacteria* on the NusG tree were well separated, and the clades inside each class showed a topology nearly identical to that of the genome tree built using 120 ubiquitous marker genes for microbial classification, bac120 (53). In contrast, the RfaH tree topology was different from that of the genome tree, suggesting that while the evolution of NusG was vertical, HGT events contributed substantially to the evolution of RfaH.

10.1128/mBio.02717-20.3 FIG S3 New RfaH model hits at the family level of *Bacteria*. The maximum-likelihood phylogenetic tree was downloaded from AnnoTree (77; http://annotree.uwaterloo.ca/). The percentage of RfaH hits was calculated for families with ≥10 genomes and are shown as bars on the outer ring. The percentages of RfaH genome hits are high in the *Gamma*- and *Alphaproteobacteria*. Download FIG S3, PDF file, 0.4 MB. Copyright © 2020 Wang et al. 2020 Wang et al. This content is distributed under the terms of the Creative Commons Attribution 4.0 International license.

10.1128/mBio.02717-20.4 FIG S4 New RfaH model hits in families of *Alphaproteobacteria* (A) and *Gammaproteobacteria* (B). The maximum-likelihood phylogenetic tree was downloaded from AnnoTree (77; http://annotree.uwaterloo.ca/). Untitled, there is currently no corresponding taxonomy. The percentages of genome hits were calculated for RfaH-containing families with ≥10 genomes. Families with >50% hits are in red and those with <50% hits in blue. Download FIG S4, PDF file, 0.5 MB. Copyright © 2020 Wang et al. 2020 Wang et al. This content is distributed under the terms of the Creative Commons Attribution 4.0 International license.

**FIG 4:**
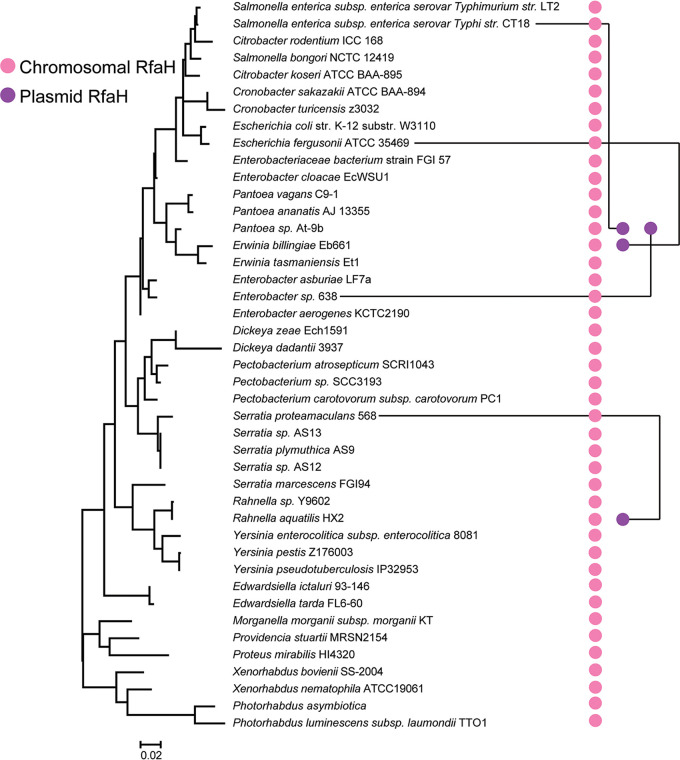
Distribution of RfaH proteins in *Enterobacteriaceae.* The maximum-likelihood phylogenetic tree was built based on sequences of the 16S rRNA genes. Chromosomal RfaH (pink) and plasmid RfaH (purple) are indicated. Plasmid-borne RfaH genes (purple dots) are connected to their best BLASTP hits among the chromosomal genes.

To study RfaH evolution in more detail, we analyzed RfaH distribution in two well-studied families of *Gammaproteobacteria*: *Enterobacteriaceae* and *Pseudomonadaceae*. Among 486 genomes of *Enterobacteriaceae*, ∼84% have RfaH. A previously defined representative genome data set of *Enterobacteriaceae* (54) was used for closer examination of RfaH distribution (Fig. 4). Among these genomes, three contained *rfaH* genes on plasmids, but the best BLAST hits of these plasmid-borne *rfaH* genes were to chromosomal genes from different strains, suggesting that RfaH can travel around on plasmids (Fig. 4). The plasmid RfaH formed a separate branch on a phylogenetic tree (Fig. S5). On the other hand, we observed similar topologies of the RfaH proteins and ribosomal trees within *Enterobacteriaceae* (Fig. 4; Fig. S5). Thus, we conclude that both vertical inheritance and HGT events shape RfaH evolution.

10.1128/mBio.02717-20.5 FIG S5 Maximum-likelihood phylogenetic tree of *Enterobacteriaceae* RfaH. The phylogenetic tree was inferred from RfaH sequences by FastTree (85) with the JTT model. The NCBI accession number of RfaH and organisms’ names are shown. The branch of plasmid RfaH is in purple. The topology of the *Enterobacteriaceae* RfaH tree is similar to that of the 16S rRNA tree of *Enterobacteriaceae* (Fig. 4). Download FIG S5, PDF file, 0.3 MB. Copyright © 2020 Wang et al. 2020 Wang et al. This content is distributed under the terms of the Creative Commons Attribution 4.0 International license.

Unlike with *Enterobacteriaceae*, in which RfaH thrives, ∼60% of *Pseudomonadaceae* lack RfaH (Fig. 2C). To reveal the origins of this different distribution, we expanded our analysis to include 617 representatives of *Pseudomonadaceae.* Most species containing RfaH are found around the root, suggesting that RfaH was present in the common ancestor and was subsequently lost in some lineages (Fig. S6A); observations that strains within the same species occasionally lose *rfaH* genes suggest that this process is ongoing (Data Set S1K). Conversely, we also observed *rfaH* duplications on the chromosome, which occurred mainly in three clades (Fig. S6B). The species of these three clades were isolated from very different environments, including sputum of a cystic fibrosis patient, cocoon mucus of an earthworm, hyperthermic compost, permafrost, plant roots, marine sediment, etc. These findings indicate that RfaH is actively evolving in *Pseudomonadaceae* through gene loss and duplication, perhaps to enable adaptation to unique ecological niches.

10.1128/mBio.02717-20.6 FIG S6 Distribution of RfaH in *Pseudomonadaceae.* (A) The midpoint-rooted maximum-likelihood phylogenetic tree was built based on 120 ubiquitous marker genes for microbial classification (53). The presence of RfaH is highlighted in purple and the ones with RfaH duplication in cyan. (B) Three clades with extensive gene duplications are shown. *P.*, *Pseudomonas*. The number of *rfaH* genes is indicated. Download FIG S6, PDF file, 0.3 MB. Copyright © 2020 Wang et al. 2020 Wang et al. This content is distributed under the terms of the Creative Commons Attribution 4.0 International license.

10.1128/mBio.02717-20.7 FIG S7 Phylogenetic comparison of NusG, RfaH, and UpxY. The numbers above colored arrows indicate the branch length sum of the longest tree path of NusG homologs. Gene duplications on the same genome are indicated with same-colored circles. Download FIG S7, PDF file, 0.8 MB. Copyright © 2020 Wang et al. 2020 Wang et al. This content is distributed under the terms of the Creative Commons Attribution 4.0 International license.

While RfaH is ubiquitous in *Proteobacteria*, we identified only one genome that encodes RfaH among 1,908 available genomes of *Bacteroidota* (*Bacteroidetes*) (Fig. 2B; Data Set S1H and I). Instead, divergent NusG^SP^ is present in approximately half of *Bacteroidota*. In Bacteroides fragilis NCTC 9343, eight UpxY proteins are encoded within different capsular polysaccharide operons (32). Each UpxY protein activates the expression of its resident operon, while the product of an adjacent *upxZ* gene interferes with the expression of heterologous *upx* operons. However, two uncharacterized UpxYs in the NCTC 9343 genome are not accompanied by UpxZ (Data Set S1L) and may perhaps act similarly to RfaH. Both the *upxY* and *rfaH* genes are present in bacteria isolated from different niches, including marine and terrestrial environments and animal hosts (Data Set S1L), and may be under pressure to rapidly adapt to changing environments. Phylogenetic comparison of NusG, RfaH, and UpxY reveals that, as judged by the average branch length, UpxY and RfaH evolve faster than NusG (Fig. S7), and both genes show extensive duplication. Thus, we conclude that NusG paralogs rapidly evolve by gene duplication and subfunctionalization.

### Steps in the molecular evolution of RfaH.

**FIG 5:**
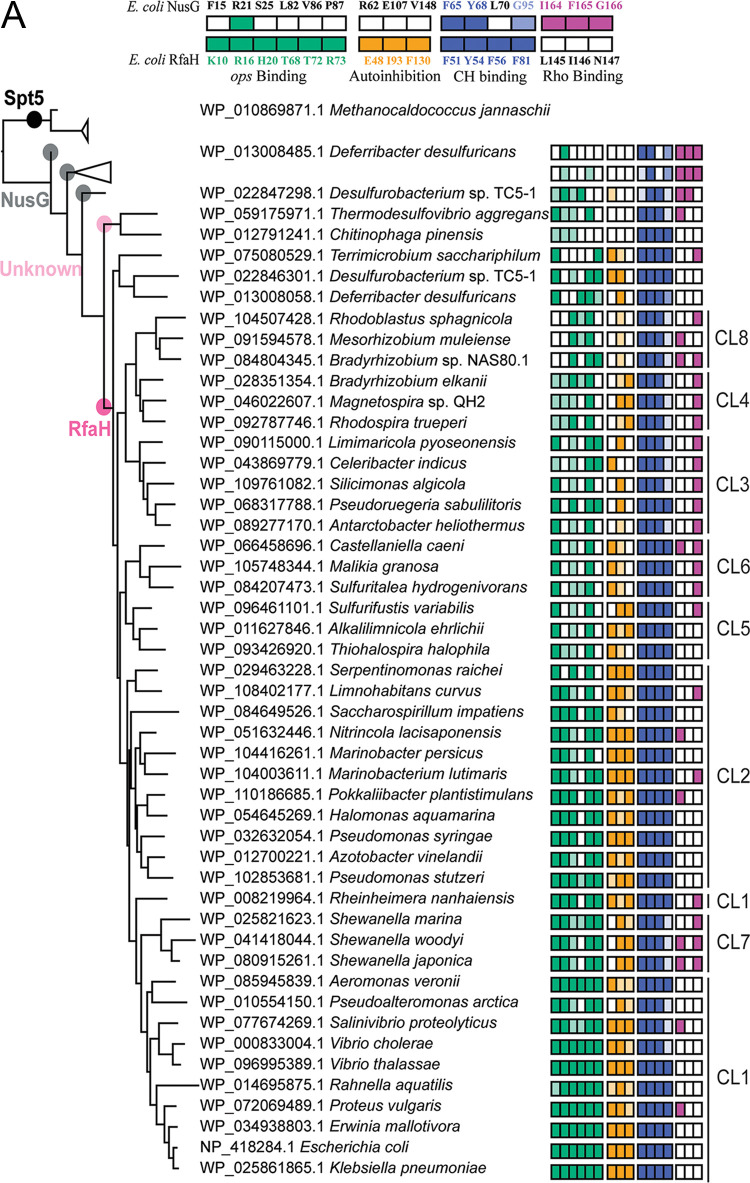

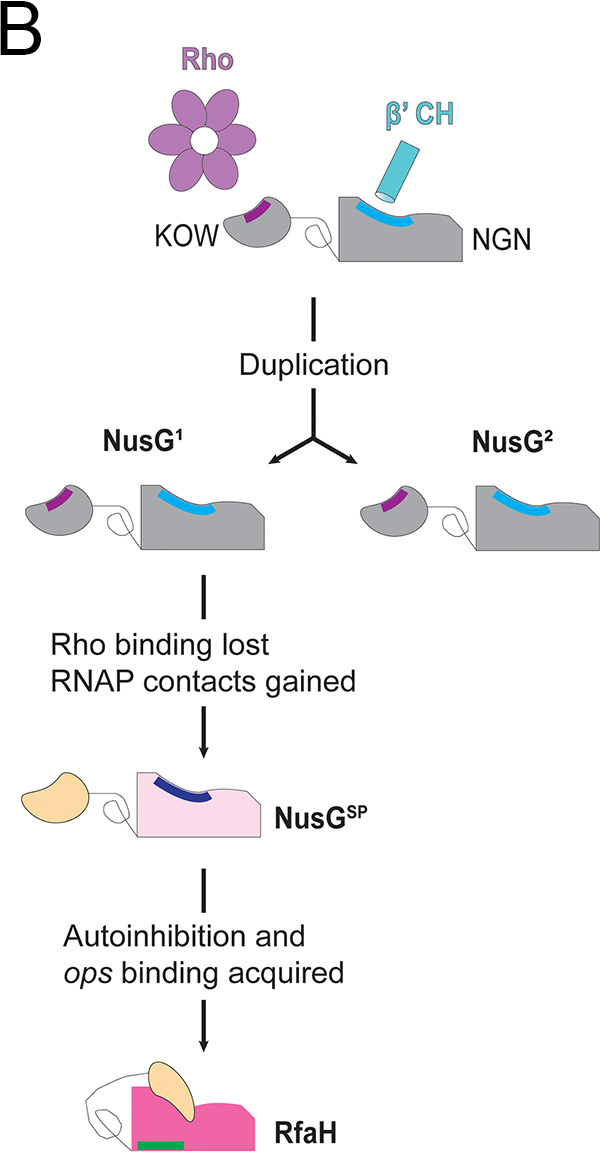
Molecular evolution of NusG and RfaH. (A) Spt5 (black), NusG (gray), unknown NusG^SP^ (light pink), and RfaH (hot pink) are marked on the maximum-likelihood phylogenetic tree. Archaeal Spt5 is used as an outgroup. NusGs with the same pattern of functional sites are collapsed. (Top) Selected functional residues in RfaH and NusG are color coded and numbered as in E. coli RfaH/NusG (NCBI accession no. NP_418284.1/NP_418409.1). Lighter colors indicate conservative substitutions. CL1 to -8 denote RfaH clusters. (B) A stepwise conversion of NusG into RfaH.

In E. coli, NusG and RfaH bind to the same site on RNAP yet have opposite effects on gene expression. NusG is abundant, essential, and acts genome-wide to aid Rho silencing of xenogenes, whereas RfaH inhibits Rho in just a few horizontally acquired operons that are dispensable for survival but necessary for virulence. Transformation of a NusG duplicate into a fully specialized RfaH protein requires several key events: (i) loss of binding to Rho, which is an essential function of NusG (43); (ii) an increased affinity for RNAP (5), which enables RfaH to compete with 100-fold more abundant NusG (28); and (iii) target-specific recruitment, which limits RfaH action to a subset of operons, thereby preventing dysregulation of NusG-controlled genes (20). Recent structural and functional analyses of E. coli NusG and RfaH identified individual residues responsible for their differences, allowing us to investigate the molecular evolution of this family (Fig. 5; Data Set S1M).

Our analysis allowed for the identification of a group of uncharacterized proteins homologous to RfaH. Phylogenetic reconstruction using Spt5 as an outgroup showed that this group of proteins and RfaH sequences are in two separate branches and that they both have NusG from *Desulfurobacterium* sp. strain TC5-1 as their common ancestor (Fig. 5A). *Desulfurobacterium* sp. TC5-1 belongs to *Aquificae*, which are thought to be among the most deeply diverging bacterial lineages, along with *Thermotogae* and *Thermodesulfobacteria* (55).

We previously proposed that the NusG paralog first lost its ability to bind Rho (Fig. 5B), most likely by altering the Rho contact residues in the NusG-KOW motif (20). Our current data support this scenario. We recently found that a conserved 5-residue loop of NusG, including residues I164, F165, and G166, makes key contacts with Rho (23); furthermore, this loop enables RfaH binding to Rho upon replacement of a loop in RfaH, which contains residues L145-I146-N147 at the corresponding positions (23). Our analysis reveals that the Rho-binding residues were lost by RfaH early on (Fig. 5A), which might be expected given that the opposite effects on Rho termination underlie cellular functions of NusG and RfaH.

Next, we envisioned that increased hydrophobicity of the NGN led to a protein with a high affinity for RNAP, which was able to compete with NusG. The RNAP β′ CH domain interacts with a hydrophobic patch on the NGNs of NusG and RfaH (5). RfaH NGN is more hydrophobic, and RfaH outcompetes NusG *in vitro* and *in vivo* (5, 20), even though NusG outnumbers RfaH 100:1 (28). RfaH residue F56 is required for binding to RNAP, and its replacement of Leu, the corresponding residue in NusG, confers binding defects (56). F56 is present in RfaH, unknown proteins, and NusG of *Desulfurobacterium* sp. TC5-1 (Fig. 5A), suggesting that stable interactions with RNAP are important for keeping RfaH in the game of evolution by preventing its displacement by a more abundant NusG. In contrast, F81 in RfaH or the corresponding G95 in NusG makes contact with RNAP in both proteins and is not highly conserved.

Finally, NusG^SP^ had to become soluble and to evolve a sequence-specific recruitment mechanism to control several targets in *trans*. In autoinhibited RfaH, the KOW domain, which is folded as an α-helical hairpin, unlike KOW domains of all other NusGs, shields a hydrophobic surface on the NGN that serves as an RNAP-binding site (29). An opposite side of the NGN contains a patch of residues that recognize the *ops* DNA (Fig. 1), which folds into a small hairpin on the RNAP surface (26). In addition to making direct contacts with the NGN, *ops* halts RNAP to facilitate RfaH recruitment (26); *ops*-like sequences induce pausing of phylogenetically diverse RNAPs (57).

Nearly all *ops* bases are required for RfaH function, and several RfaH residues directly contact the *ops* DNA hairpin (5, 26). We reason that such a complex mechanism must have evolved incrementally, perhaps with NusG^SP^ initially binding to a paused RNAP and then learning to recognize DNA. Mapping of the RfaH DNA-binding determinants on the phylogenetic tree (Fig. 5A) is consistent with a sequential acquisition of residues that bind DNA: K10 (F in NusG) acquisition preceded the emergence of RfaH, whereas R73 arose later.

We believe that autoinhibition controls RfaH recruitment indirectly, by making RfaH binding to RNAP dependent on the presence of the *ops* signal. RfaH residues E48, I93, and F130 are required for autoinhibition; their replacement allows sequence-independent, NusG-like recruitment of RfaH (27, 58). RfaH contacts with the *ops*-paused complex relieve autoinhibition, exposing the RNAP-binding site on the NGN (30). The acquisition of residues that mediate interdomain interactions coincide with that of the DNA-binding residues (Fig. 5A), consistent with autoinhibition and *ops* contacts acting in concert. In summary, our analysis supports a sequential transformation of NusG into RfaH in which the exclusion of Rho binding and increased binding to RNAP precede sequence-specific recruitment to the elongation complex (Fig. 5B).

### RfaH targets and gene neighbors.

**FIG 6:**
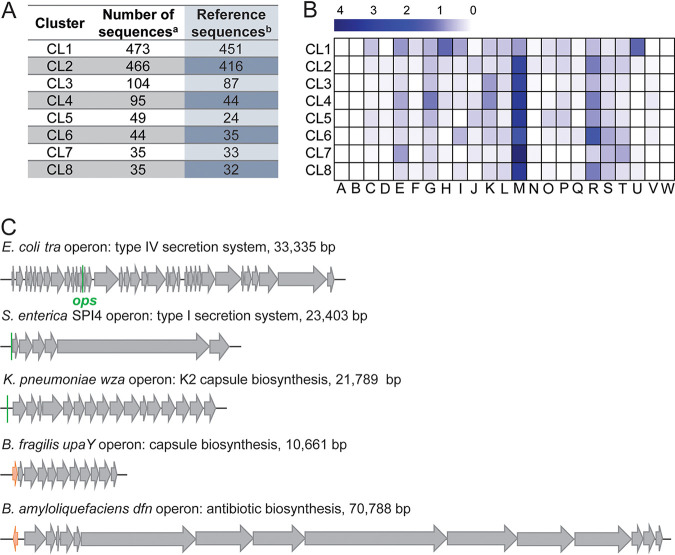
RfaH clusters, genomic contexts, and targets. (A) The eight clusters. Footnote a, RfaHs found in GTDB_reps were clustered into eight clusters (Data Set S1H and N). The number of total sequences of different clusters are presented. Footnote b, a subset of different CLs containing NCBI reference sequences only. The number of sequences is shown. (B) Heatmap showing distribution of COG functional categories (represented by A to W) of RfaH neighbor genes; there are five genes on each side. The number of genes in every COG category was normalized by the number of RfaH reference sequences. (C) Operons activated by enterobacterial RfaHs and other NusG^SP^ proteins; positions of *ops* sites (green) and NusG^SP^ genes (orange) are shown. COG categories can be accessed at https://www.ncbi.nlm.nih.gov/COG/.

While E. coli RfaH is monocistronic and acts in *trans*, other NusG^SP^ proteins, such as Myxococcus xanthus TaA (33) and UpxY (32), are encoded within their target operons. We wondered whether RfaH-like proteins, which display significant variations in their functional regions (Fig. 5A), could fall into different groups, perhaps associated with particular regulatory contexts. Markov clustering of all RfaH sequences identified in this study revealed eight distinct clusters, CL1 to CL8 (Fig. 6A; Fig. S8; Data Set S1N). Using TREND (40), we found that, unlike with the invariant gene neighborhood of *nusG* (see above), the gene neighbors of *rfaH* were highly diverse; they encoded polysaccharide biosynthesis enzymes, nucleoid-associated protein H-NS, toxin-antitoxin systems, secondary metabolites, Tat protein secretion system, etc.

10.1128/mBio.02717-20.8 FIG S8 Sequence logos of eight RfaH clusters (CL). The logos were created based on multiple-sequence alignments of RfaH sequences from each CL using WebLogo (G. E. Crooks, G. Hon, J. M. Chandonia, and S. E. Brenner, Genome Res 14:1188–1190, 2004; https://weblogo.berkeley.edu/). The logos were trimmed to begin with the conserved residue tryptophan in all CLs. Functional sites discussed in Fig. 5A are marked with colored squares. Green, *ops* binding sites; orange, sites responsible for autoinhibition; blue, RNAP β′ CH-binding sites; purple, Rho-binding sites. Download FIG S8, PDF file, 0.7 MB. Copyright © 2020 Wang et al. 2020 Wang et al. This content is distributed under the terms of the Creative Commons Attribution 4.0 International license.

To assess whether each cluster could be associated with a subset of genes, we assigned their gene neighbors to cluster of orthologous group (COG) categories (Fig. 6B) (38). Similarly to E. coli RfaH, which is included in CL1, RfaHs of CL1 were not strongly associated with a particular COG category, although H (coenzyme metabolism) and U (secretion) genes were frequent. These diffuse-pattern proteins act in *trans* on distant targets. In contrast, genes involved in cell envelope biogenesis (M), which are known targets of NusG^SP^ regulators, were overrepresented among neighbors of CL2 to CL8; glycosyltransferases, nucleoside-diphosphate-sugar epimerases, and exopolysaccharide biosynthesis functions were most common (Fig. 6B; Fig. S9A). Notable differences exist among these clusters (Fig. 6B; Fig. S9A). CL1 is frequently adjacent to Sec-independent protein secretion pathway functions (U). CL4 is associated with a helix-turn-helix (HTH) transcriptional regulator (K). CL6 neighbors encode undecaprenyl pyrophosphate synthase, involved in terpenoid biosynthesis (I), and nucleoid-associated protein H-NS (R), whereas CL7 comprises a group of diverse RfaHs from *Shewanella* that are encoded within putative exopolysaccharide operons (Fig. S9B), an arrangement resembling B. fragilis operons controlled by diverse UpxY proteins (32). Many CL7 genes are adjacent to signal transduction (CheY) and envelope biogenesis (ABC transporter) genes, but their relative orientations differ among CL7 members.

10.1128/mBio.02717-20.9 FIG S9 Enrichment of neighboring genes of RfaH clusters (CL). (A) A total of 1,054 COGs were assigned to neighbor genes of *rfaH* (five genes on both sides of *rfaH* genes). Every circle represents a COG. The percentage of COG was calculated as (raw count of COG)/(total neighbor genes of one CL). Then, COGs were assigned with a unique integer in the range of 1 to 1,054 and the same COG in different CLs will be assigned with the same integer. These integers were used to build the *x* axis. The identities of highly abundant COGs (38) are indicated. (B) Example of the conversed CL7 *rfaH* location. From left to right, white arrows indicate chemotaxis signaling genes, light brown arrows indicate a relationship to type III secretion systems, black arrows indicate a transport system, brown arrows indicate a major facilitator superfamily (MFS) transporter, hot pink arrows indicate *rfaH*, green bars indicate the *ops* site, and gray arrows indicate an exopolysaccharide operon. Download FIG S9, PDF file, 0.3 MB. Copyright © 2020 Wang et al. 2020 Wang et al. This content is distributed under the terms of the Creative Commons Attribution 4.0 International license.

In addition to activating several chromosomal targets, RfaH activates an F plasmid *tra* operon, which encodes a type IV secretion system (Fig. 6C) and is required for conjugation (59). Other plasmids encode resident NusG^SP^s in their *tra* operons. As we await experimental assessment of their functions, this genetic syntax suggests that plasmid NusG^SP^ acts as an antiterminator of *tra* operons, which are among the longest bacterial operons and are thus expected to be prone to premature termination. Carrying a resident antiterminator confers a significant advantage to plasmids that, unlike F, are transferred between different species. Conjugative plasmids are major contributors toward the clinical dissemination of antibiotic resistance, and some of these plasmids encode NusG^SP^s (60, 61).

RfaH and other NusG^SP^s are required for the expression of very diverse macromolecules, including adhesins, antibiotics, capsular polysaccharides, toxins, etc. The most obvious common feature of NusG^SP^ targets is their length (Fig. 6C). A shared ability of all NusG-like proteins to make RNA synthesis more efficient suggests a mechanism in which NusG^SP^-bound RNAP ignores intragenic termination signals; consistently, NusG^SP^ is annotated as an antiterminator. However, while RfaH increases gene expression hundreds of folds, its antitermination activity makes only a minor contribution to its effects *in vivo* (62). Instead, RfaH excludes NusG from RNAP and promotes ribosome recruitment, thereby inhibiting premature RNA release by Rho (27). Furthermore, by coupling RNAP to the ribosome (27), RfaH may enable the complete synthesis of long polypeptides, such as a giant 5,559-amino-acid-long nonfimbrial adhesin encoded by *Salmonella* pathogenicity island IV (63) (Fig. 6C). Similarly, LoaP-like regulators (31) may promote translation of 4,200- and 5,200-amino-acid-long polyketide synthases in the Bacillus amyloliquefaciens *dfn* operon.

The marked diversity of their gene neighborhoods supports a view that RfaH-like regulators act on any operon, once recruited; indeed, E. coli and Klebsiella pneumoniae RfaH activate expression of the Photorhabdus luminescens *lux* operon, as long as the *ops* element is present in the leader region (64). However, in this work, we show that different types of RfaH-like proteins are associated with different classes of neighbors (Fig. 6B), a correlation that may reflect their evolutionary history or distinct mechanisms of recruitment. E. coli RfaH is the only representative for which a detailed mode of recruitment is known, and future studies are required to address this question.

### Concluding remarks.

The only ubiquitous family of transcription factors comprises two very different classes of regulators. One class includes essential general elongation factors that coevolved with RNAP since the LUCA (1). These NusG-like core regulators are recruited to RNAP once it escapes from a promoter, replacing transcription initiation factors that bind to the same site (18, 19), and remain associated with RNAP transcribing all genes (20, 65). Here, we show that the bacterial NusG protein is present in genomes of all cells that utilize bacterial RNAPs, except a few endosymbionts and some algae. What makes NusG indispensable?

Although their sequences have diverged considerably, bacterial, archaeal, and eukaryal factors make remarkably similar interactions with RNAP that are thought to increase the enzyme’s processivity, acting akin to replicative clamps (66); the NGNs are necessary and sufficient for RNAP modifications (14, 29, 67). This antitermination function of NusG, reflected in genome annotations, has long been thought to be its signature activity. However, NusG alone has only modest effects on RNA synthesis (9). Instead, antitermination is achieved through the assembly of large nucleoprotein complexes, e.g., on bacteriophage λ RNA, in which the NusG-KOW domain makes contact with diverse protein partners (10). In fact, it is through alternative contacts with Rho (23) or ribosome (21) that the NusG-KOW domain determines the fate of the nascent RNA. Multiple Spt5 KOW domains play analogous functions in eukaryotes, coupling RNA synthesis to splicing, polyadenylation, and other cotranscriptional processes (3). Transcription of chloroplast genomes by PEP depends on its binding to several accessory proteins (68), including NusG (45). We speculate that the NusG-KOW domain acts as a hub for PEP complex assembly.

Despite its ubiquity, NusG is a dissociable factor rather than an RNAP subunit, a property exploited by the second class of NusG proteins exemplified by RfaH. These regulators outcompete NusG for binding to RNAP and exert much stronger antitermination effects (5) but must be selectively recruited to only a few targets to avoid misregulation of housekeeping genes (20). In the case of RfaH, targeted recruitment is achieved through a complex DNA-dependent mechanism (26). Here, we show that RfaH-like proteins are rapidly evolving through a combination of HGT and vertical inheritance. We identified eight distinct groups of RfaH that we propose control different sets of genes, sometimes coevolving with their targets. While the RfaH-NGN mediates recruitment to RNAP and DNA, we hypothesize that the RfaH-KOW domain plays key regulatory roles. The KOW domain controls RfaH recruitment indirectly, through autoinhibition (58), is thought to load the ribosome onto mRNA lacking ribosome-binding sites (27), and may interact with some membrane components during secretion of proteins whose expression it activates (69). While RfaH is not strictly essential for growth in the lab, it is critical for expression of the cell wall, capsules, adhesins, siderophores, and conjugative pili, whereas other NusG^SP^s are essential for the synthesis of capsules and antibiotics (7), molecules that determine bacterial success in natural environments.

Eukaryotes also encode multiple copies of Spt5 (Fig. 2A), and specialized paralogs have been implicated in the regulation of RNA silencing and meiosis (34, 70). Thus, all life depends on the NusG-like regulators to balance the expression of housekeeping genes with niche-specific demands. The mechanisms by which this balance is maintained remain to be elucidated.

## MATERIALS AND METHODS

Taxonomy information used in this study was derived from the Genome Taxonomy Database (GTDB; https://gtdb.ecogenomic.org/) (39). Archaeplastida, Chromista, and Plantae are artificial groups (71–73) and used solely for brevity in this paper.

### Construction of a new RfaH model.

RfaH (NCBI accession no. NP_418284.1) from Escherichia coli strain K-12 substrain MG1655 was used as a query in BLAST searches against genomes of selected representatives to find potential RfaH homologs. One species from each family of *Proteobacteria* was selected as a representative. All potential RfaH sequences were verified using a reciprocal best BLAST hit approach (74) (see Fig. S2 in the supplemental material for an example). The final set of 103 RfaH sequences was used to construct an initial multiple-sequence alignment (MSA). Based on the MSA, an initial HMM profile was generated and used to query the UniProt Reference Proteomes database (v. 2019-09). The hits were filtered based on known conserved positions in RfaH and structural information to collect an extended set of RfaH protein sequences. The redundancy of the set was reduced to the 80% identity level by CD-HIT, and a new MSA was generated based on the reduced sequence set. This set was used to generate a final HMM profile. The final profile was used to query the UniProt reference proteome database and to set the trusted and noise cutoffs of the profile.

### Database of species representatives (GTDB_reps).

The list of species representatives of bacteria and archaea (release 89.0) was downloaded from the GTDB (39). The genome files (file type: protein FASTA) were retrieved from NCBI using Batch Entrez (https://www.ncbi.nlm.nih.gov/sites/batchentrez). A total of 18,436 bacterial genome files and 847 archaeal genome files were downloaded and used as a database of species representatives in this study, which was named GTDB_reps.

### Distribution of NusG and RfaH.

NusG TIGRfam and the newly built RfaH HHM were used to search against GTDB_reps by HMMER (75). Taxonomy assignment of the collected protein sequences was done using a custom python script. The percentage of genome hits was calculated using a custom python script. The results were visualized on phylogenetic trees by FigTree (76). The maximum-likelihood genome trees were downloaded from AnnoTree (77; http://annotree.uwaterloo.ca/).

### Identification of NusG in Eukaryota.

We used the NusG protein sequence (NCBI accession no. WP_012415655.1) from Elusimicrobium minutum to search eukaryotic protein databases. We used BLASTP and PSI-BLAST against the nonredundant database at the NCBI and a BLASTP search against the oneKP database (78), with default parameters (May 2020). Domain identification was carried out using the TREND (40) and HHpred (79) servers. Multiple-sequence alignments were constructed using the L-INS-I algorithm of MAFFT (80) and edited in Jalview (81). A maximum-likelihood phylogenetic tree was constructed using the MEGA X package (82) and edited in the Interactive Tree of Life (iTOL) v4 tool (83).

### RfaH evolution events.

To study the topology of NusG and RfaH phylogenetic trees, representatives were selected from GTDB_reps (Data Set S1J). One representative genome containing both NusG and RfaH was selected from each family. A total of 82 family representatives of *Proteobacteria* were selected. A maximum-likelihood bacterial genome tree of family representatives was inferred from a concatenated alignment of 120 ubiquitous single-copy proteins, also known as the bac120 data set (53) using RAxML (84). Maximum-likelihood phylogenetic trees of NusG and RfaH were constructed using FastTree (85) and RAxML (84). The trees constructed by the two methods showed similar topologies. To show examples of evolution events, two families, *Enterobacteriaceae* and *Pseudomonadaceae*, were investigated. The maximum-likelihood phylogenetic tree of 16S rRNA sequences of *Enterobacteriaceae* was from a previous study (54), whereas a maximum-likelihood genome tree of *Pseudomonadaceae* was inferred from the bac120 data set. The presence of RfaH was determined using the new RfaH model. The maximum-likelihood RfaH tree of *Enterobacteriaceae* was inferred using FastTree (85).

### Phylogenetic tree for molecular evolution study.

To study the molecular evolution of RfaH, a data set was compiled with three parts (Data Set S1M). The first part was representative genomes containing both RfaH and NusG. To select these representatives, a maximum-likelihood phylogenetic tree was inferred from 1,922 RfaH sequences (Data Set S1H) by FastTree (85). Then representatives were selected from this phylogenetic tree according to tree depth. The second part was representative genomes containing proteins which have bit scores between trusted and noise cutoffs of the new RfaH model (referred to as unknown NusG^SP^s). The third part was representative archaeal genomes containing Spt5, which served as an outgroup. The structural alignment was performed with MAFFT-DASH (86). The maximum-likelihood phylogenetic tree was inferred using FastTree with the JTT model (85) and RAxML with the LG4X model (84). The two programs produced nearly identical phylogenetic trees.

### Clustering of RfaH protein sequences.

RfaH protein sequences collected running the new RfaH HMM profile against GTDB_reps were clustered in a stepwise fashion:

Step 1 reduced the redundancy of the sequences at a 95% identity level, giving a final set of 1,481 sequences.

In step 2, reciprocal BLASTP all-vs-all was run using the final set. With the result, an undirected graph was built. The following cutoffs were used to construct the graph edges: an E value less than or equal to 5e–30 and a coverage of ≥80%. The edge weights were initialized using an average of two E values of each reciprocal BLASTP. Using this graph, Markov clustering was performed. An inflation value of 5 was used, as it gave the most efficient clustering. The majority of the sequences ended up in eight coherent clusters.

### Neighbor genes of RfaH.

Gene neighborhoods of 1,122 reference *rfaH* genes (Fig. 6A) were determined using TREND (40); each neighbor gene was assigned to clusters of orthologous groups (COGs) (38, 87). The distribution of COGs in the eight RfaH clusters were presented by Heatmap using the R package (http://www.R-project.org/).

### UpxY search.

BLASTP with the E value threshold of <10^−10^ was used to query GTDB_reps with eight UpxY protein sequences from B. fragilis NCTC 9343 (32). Representatives were selected to build a maximum-likelihood phylogenetic tree with RfaH and NusG (Data Set S1L). The structural alignment computed by MAFFT-DASH (86) was used to build the phylogenetic tree. The phylogenetic tree was inferred using FastTree with the JTT model (85).

### NusG family detection.

An entire list of GTDB genome identifiers (release 89.0) was downloaded. Based on the list, 129,663 genomes were fetched from the NCBI and compiled into a complete database. The database was searched using profile HMMs of eight ubiquitous vertically inherited proteins: NusG, SecE, RecA, L1, L5, L6, S2, and S7.

### Software.

We used the following software: AnnoTree v1.2.0 (77), CD-HIT v4.7 (88), FastTree v2.1.10 (85), FigTree v1.4.4 (76), HMMER Web server v2.40.0 (36), HMMER package v3.3 (75), Jalview v2.11.0 (81), MAFFT v7.450 (89), NCBI BLAST 2.9.0+ (90), Python 3.8.2 (91), RAxML v8.2.12 (84), and R 3.6.2 (92). Python codes used in this study are available upon request.

### Models.

Models were the new RfaH HMM (this study; to be included in the MiST database [37]), RfaH TIGRfam (TIGR01955), NusG Pfam (PF02357), NusG TIGRfam (TIGR00922), Spt5-NGN Pfam (PF03439), NusA_N Pfam (PF08529), SecE Pfam (PF00584), RecA Pfam (PF00154), L1 Pfam (PF00687), L5 Pfam (PF00281), L6 Pfam (PF00347), S2 Pfam (PF00318), and S7 Pfam (PF00177).

## References

[1] Werner F 2012 A nexus for gene expression—molecular mechanisms of Spt5 and NusG in the three domains of life. J Mol Biol 417:13–27. doi:10.1016/j.jmb.2012.01.031. 22306403 PMC3382729

[2] Steiner T, Kaiser JT, Marinkovic S, Huber R, Wahl MC 2002 Crystal structures of transcription factor NusG in light of its nucleic acid- and protein-binding activities. EMBO J 21:4641–4653. doi:10.1093/emboj/cdf455. 12198166 PMC126194

[3] Hartzog GA, Fu J 2013 The Spt4-Spt5 complex: a multi-faceted regulator of transcription elongation. Biochim Biophys Acta 1829:105–115. doi:10.1016/j.bbagrm.2012.08.007. 22982195 PMC3545043

[4] Ehara H, Yokoyama T, Shigematsu H, Yokoyama S, Shirouzu M, Sekine SI 2017 Structure of the complete elongation complex of RNA polymerase II with basal factors. Science 357:921–924. doi:10.1126/science.aan8552. 28775211

[5] Kang JY, Mooney RA, Nedialkov Y, Saba J, Mishanina TV, Artsimovitch I, Landick R, Darst SA 2018 Structural basis for transcript elongation control by NusG family universal regulators. Cell 173:1650–1662.e14. doi:10.1016/j.cell.2018.05.017. 29887376 PMC6003885

[6] Klein BJ, Bose D, Baker KJ, Yusoff ZM, Zhang X, Murakami KS 2011 RNA polymerase and transcription elongation factor Spt4/5 complex structure. Proc Natl Acad Sci U S A 108:546–550. doi:10.1073/pnas.1013828108. 21187417 PMC3021056

[7] Artsimovitch I, Knauer SH 2019 Ancient transcription factors in the news. mBio 10:e01547-18. doi:10.1128/mBio.01547-18. PMC6391919 30808693

[8] Vos SM, Farnung L, Urlaub H, Cramer P 2018 Structure of paused transcription complex Pol II-DSIF-NELF. Nature 560:601–606. doi:10.1038/s41586-018-0442-2. 30135580 PMC6245578

[9] Herbert KM, Zhou J, Mooney RA, Porta AL, Landick R, Block SM 2010 E. coli NusG inhibits backtracking and accelerates pause-free transcription by promoting forward translocation of RNA polymerase. J Mol Biol 399:17–30. doi:10.1016/j.jmb.2010.03.051. 20381500 PMC2875378

[10] Krupp F, Said N, Huang YH, Loll B, Burger J, Mielke T, Spahn CMT, Wahl MC 2019 Structural basis for the action of an all-purpose transcription anti-termination factor. Mol Cell 74:143–157.e5. doi:10.1016/j.molcel.2019.01.016. 30795892

[11] Guo M, Xu F, Yamada J, Egelhofer T, Gao Y, Hartzog GA, Teng M, Niu L 2008 Core structure of the yeast spt4-spt5 complex: a conserved module for regulation of transcription elongation. Structure 16:1649–1658. doi:10.1016/j.str.2008.08.013. 19000817 PMC2743916

[12] Wenzel S, Martins BM, Rosch P, Wohrl BM 2009 Crystal structure of the human transcription elongation factor DSIF hSpt4 subunit in complex with the hSpt5 dimerization interface. Biochem J 425:373–380. doi:10.1042/BJ20091422. 19860741

[13] Drogemuller J, Stegmann CM, Mandal A, Steiner T, Burmann BM, Gottesman ME, Wohrl BM, Rosch P, Wahl MC, Schweimer K 2013 An autoinhibited state in the structure of Thermotoga maritima NusG. Structure 21:365–375. doi:10.1016/j.str.2012.12.015. 23415559 PMC3764593

[14] Hirtreiter A, Damsma GE, Cheung AC, Klose D, Grohmann D, Vojnic E, Martin AC, Cramer P, Werner F 2010 Spt4/5 stimulates transcription elongation through the RNA polymerase clamp coiled-coil motif. Nucleic Acids Res 38:4040–4051. doi:10.1093/nar/gkq135. 20197319 PMC2896526

[15] Sanders TJ, Lammers M, Marshall CJ, Walker JE, Lynch ER, Santangelo TJ 2019 TFS and Spt4/5 accelerate transcription through archaeal histone-based chromatin. Mol Microbiol 111:784–797. doi:10.1111/mmi.14191. 30592095 PMC6417941

[16] Martinez-Rucobo FW, Sainsbury S, Cheung AC, Cramer P 2011 Architecture of the RNA polymerase-Spt4/5 complex and basis of universal transcription processivity. EMBO J 30:1302–1310. doi:10.1038/emboj.2011.64. 21386817 PMC3094117

[17] Turtola M, Belogurov GA 2016 NusG inhibits RNA polymerase backtracking by stabilizing the minimal transcription bubble. Elife 5:e18096. doi:10.7554/eLife.18096. 27697152 PMC5100998

[18] Grohmann D, Nagy J, Chakraborty A, Klose D, Fielden D, Ebright RH, Michaelis J, Werner F 2011 The initiation factor TFE and the elongation factor Spt4/5 compete for the RNAP clamp during transcription initiation and elongation. Mol Cell 43:263–274. doi:10.1016/j.molcel.2011.05.030. 21777815 PMC3223566

[19] Sevostyanova A, Svetlov V, Vassylyev DG, Artsimovitch I 2008 The elongation factor RfaH and the initiation factor sigma bind to the same site on the transcription elongation complex. Proc Natl Acad Sci U S A 105:865–870. doi:10.1073/pnas.0708432105. 18195372 PMC2242686

[20] Belogurov GA, Mooney RA, Svetlov V, Landick R, Artsimovitch I 2009 Functional specialization of transcription elongation factors. EMBO J 28:112–122. doi:10.1038/emboj.2008.268. 19096362 PMC2634734

[21] Burmann BM, Schweimer K, Luo X, Wahl MC, Stitt BL, Gottesman ME, Rösch P 2010 A NusE:NusG complex links transcription and translation. Science 328:501–504. doi:10.1126/science.1184953. 20413501

[22] Saxena S, Myka KK, Washburn R, Costantino N, Court DL, Gottesman ME 2018 Escherichia coli transcription factor NusG binds to 70S ribosomes. Mol Microbiol 108:495–504. doi:10.1111/mmi.13953. 29575154 PMC5980749

[23] Lawson MR, Ma W, Bellecourt MJ, Artsimovitch I, Martin A, Landick R, Schulten K, Berger JM 2018 Mechanism for the regulated control of bacterial transcription termination by a universal adaptor protein. Mol Cell 71:911–922.e4. doi:10.1016/j.molcel.2018.07.014. 30122535 PMC6151137

[24] Mitra P, Ghosh G, Hafeezunnisa M, Sen R 2017 Rho protein: roles and mechanisms. Annu Rev Microbiol 71:687–709. doi:10.1146/annurev-micro-030117-020432. 28731845

[25] Artsimovitch I, Landick R 2002 The transcriptional regulator RfaH stimulates RNA chain synthesis after recruitment to elongation complexes by the exposed nontemplate DNA strand. Cell 109:193–203. doi:10.1016/s0092-8674(02)00724-9. 12007406

[26] Zuber PK, Artsimovitch I, NandyMazumdar M, Liu Z, Nedialkov Y, Schweimer K, Rösch P, Knauer SH 2018 The universally-conserved transcription factor RfaH is recruited to a hairpin structure of the non-template DNA strand. Elife 7:e36349. doi:10.7554/eLife.36349. 29741479 PMC5995543

[27] Burmann BM, Knauer SH, Sevostyanova A, Schweimer K, Mooney Rachel A, Landick R, Artsimovitch I, Rösch P 2012 An α helix to β barrel domain switch transforms the transcription factor RfaH into a translation factor. Cell 150:291–303. doi:10.1016/j.cell.2012.05.042. 22817892 PMC3430373

[28] Schmidt A, Kochanowski K, Vedelaar S, Ahrné E, Volkmer B, Callipo L, Knoops K, Bauer M, Aebersold R, Heinemann M 2016 The quantitative and condition-dependent Escherichia coli proteome. Nat Biotechnol 34:104–110. doi:10.1038/nbt.3418. 26641532 PMC4888949

[29] Belogurov GA, Vassylyeva MN, Svetlov V, Klyuyev S, Grishin NV, Vassylyev DG, Artsimovitch I 2007 Structural basis for converting a general transcription factor into an operon-specific virulence regulator. Mol Cell 26:117–129. doi:10.1016/j.molcel.2007.02.021. 17434131 PMC3116145

[30] Zuber PK, Schweimer K, Rosch P, Artsimovitch I, Knauer SH 2019 Reversible fold-switching controls the functional cycle of the antitermination factor RfaH. Nat Commun 10:702. doi:10.1038/s41467-019-08567-6. 30742024 PMC6370827

[31] Goodson JR, Klupt S, Zhang C, Straight P, Winkler WC 2017 LoaP is a broadly conserved antiterminator protein that regulates antibiotic gene clusters in Bacillus amyloliquefaciens. Nat Microbiol 2:17003. doi:10.1038/nmicrobiol.2017.3. 28191883 PMC5913657

[32] Chatzidaki-Livanis M, Weinacht KG, Comstock LE 2010 Trans locus inhibitors limit concomitant polysaccharide synthesis in the human gut symbiont Bacteroides fragilis. Proc Natl Acad Sci U S A 107:11976–11980. doi:10.1073/pnas.1005039107. 20547868 PMC2900635

[33] Paitan Y, Orr E, Ron EZ, Rosenberg E 1999 A NusG-like transcription anti-terminator is involved in the biosynthesis of the polyketide antibiotic TA of Myxococcus xanthus. FEMS Microbiol Lett 170:221–227. doi:10.1111/j.1574-6968.1999.tb13377.x. 9919671

[34] Bies-Etheve N, Pontier D, Lahmy S, Picart C, Vega D, Cooke R, Lagrange T 2009 RNA-directed DNA methylation requires an AGO4-interacting member of the SPT5 elongation factor family. EMBO Rep 10:649–654. doi:10.1038/embor.2009.31. 19343051 PMC2711833

[35] El-Gebali S, Mistry J, Bateman A, Eddy SR, Luciani A, Potter SC, Qureshi M, Richardson LJ, Salazar GA, Smart A, Sonnhammer ELL, Hirsh L, Paladin L, Piovesan D, Tosatto SCE, Finn RD 2019 The Pfam protein families database in 2019. Nucleic Acids Res 47:D427–D432. doi:10.1093/nar/gky995. 30357350 PMC6324024

[36] Potter SC, Luciani A, Eddy SR, Park Y, Lopez R, Finn RD 2018 HMMER web server: 2018 update. Nucleic Acids Res 46:W200–W204. doi:10.1093/nar/gky448. 29905871 PMC6030962

[37] Gumerov VM, Ortega DR, Adebali O, Ulrich LE, Zhulin IB 2020 MiST 3.0: an updated microbial signal transduction database with an emphasis on chemosensory systems. Nucleic Acids Res 48:D459–D464. doi:10.1093/nar/gkz988. 31754718 PMC6943060

[38] Galperin MY, Makarova KS, Wolf YI, Koonin EV 2015 Expanded microbial genome coverage and improved protein family annotation in the COG database. Nucleic Acids Res 43:D261–D269. doi:10.1093/nar/gku1223. 25428365 PMC4383993

[39] Parks DH, Chuvochina M, Waite DW, Rinke C, Skarshewski A, Chaumeil P-A, Hugenholtz P 2018 A standardized bacterial taxonomy based on genome phylogeny substantially revises the tree of life. Nat Biotechnol 36:996–1004. doi:10.1038/nbt.4229. 30148503

[40] Gumerov VM, Zhulin IB 2020 TREND: a platform for exploring protein function in prokaryotes based on phylogenetic, domain architecture and gene neighborhood analyses. Nucleic Acids Res 48:W72–W76. doi:10.1093/nar/gkaa243. 32282909 PMC7319448

[41] Minakhin L, Bhagat S, Brunning A, Campbell EA, Darst SA, Ebright RH, Severinov K 2001 Bacterial RNA polymerase subunit omega and eukaryotic RNA polymerase subunit RPB6 are sequence, structural, and functional homologs and promote RNA polymerase assembly. Proc Natl Acad Sci U S A 98:892–897. doi:10.1073/pnas.98.3.892. 11158566 PMC14680

[42] Ingham CJ, Dennis J, Furneaux PA 1999 Autogenous regulation of transcription termination factor Rho and the requirement for Nus factors in Bacillus subtilis. Mol Microbiol 31:651–663. doi:10.1046/j.1365-2958.1999.01205.x. 10027981

[43] Cardinale CJ, Washburn RS, Tadigotla VR, Brown LM, Gottesman ME, Nudler E 2008 Termination factor Rho and its cofactors NusA and NusG silence foreign DNA in E. coli. Science 320:935–938. doi:10.1126/science.1152763. 18487194 PMC4059013

[44] Leebens-Mack JH, Barker MS, Carpenter EJ, Deyholos MK, Gitzendanner MA, Graham SW, Grosse I, Li Z, Melkonian M, Mirarab S, Porsch M, Quint M, Rensing SA, Soltis DE, Soltis PS, Stevenson DW, Ullrich KK, Wickett NJ, DeGironimo L, Edger PP, Jordon-Thaden IE, Liu T, Melkonian B, Miles NW, Pokorny L, Quigley C, Thomas P, Villarreal JC, Augustin MM, Barrett MD, Baucom RS, Beerling DJ, Benstein RM, Biffin E, Brockington SF, Burge DO, Burris JN, Burris KP, Burtet-Sarramegna V, Caicedo AL, Cannon SB, Çebi Z, Chang Y, Chater C, Cheeseman JM, Chen T, Clarke ND, Clayton H, Covshoff S, 2019 One thousand plant transcriptomes and the phylogenomics of green plants. Nature 574:679–685. doi:10.1038/s41586-019-1693-2. 31645766 PMC6872490

[45] Pfalz J, Liere K, Kandlbinder A, Dietz KJ, Oelmüller R 2006 pTAC2, -6, and -12 are components of the transcriptionally active plastid chromosome that are required for plastid gene expression. Plant Cell 18:176–197. doi:10.1105/tpc.105.036392. 16326926 PMC1323492

[46] Yang Z, Li M, Sun Q 2020 RHON1 co-transcriptionally resolves R-loops for Arabidopsis chloroplast genome maintenance. Cell Rep 30:243–256.e5. doi:10.1016/j.celrep.2019.12.007. 31914390

[47] Emanuelsson O, Nielsen H, von Heijne G 1999 ChloroP, a neural network-based method for predicting chloroplast transit peptides and their cleavage sites. Protein Sci 8:978–984. doi:10.1110/ps.8.5.978. 10338008 PMC2144330

[48] Smith DR, Keeling PJ 2016 Protists and the wild, wild West of gene expression: new frontiers, lawlessness, and misfits. Annu Rev Microbiol 70:161–178. doi:10.1146/annurev-micro-102215-095448. 27359218

[49] Sanita Lima M, Smith DR 2017 Pervasive transcription of mitochondrial, plastid, and nucleomorph genomes across diverse plastid-bearing species. Genome Biol Evol 9:2650–2657. doi:10.1093/gbe/evx207. 29048528 PMC5737562

[50] Nowack EC, Melkonian M, Glöckner G 2008 Chromatophore genome sequence of Paulinella sheds light on acquisition of photosynthesis by eukaryotes. Curr Biol 18:410–418. doi:10.1016/j.cub.2008.02.051. 18356055

[51] Kim S, Park MG 2016 Paulinella longichromatophora sp. nov., a new marine photosynthetic testate amoeba containing a chromatophore. Protist 167:1–12. doi:10.1016/j.protis.2015.11.003. 26709891

[52] Marin B, Nowack EC, Glöckner G, Melkonian M 2007 The ancestor of the Paulinella chromatophore obtained a carboxysomal operon by horizontal gene transfer from a Nitrococcus-like gamma-proteobacterium. BMC Evol Biol 7:85. doi:10.1186/1471-2148-7-85. 17550603 PMC1904183

[53] Parks DH, Rinke C, Chuvochina M, Chaumeil P-A, Woodcroft BJ, Evans PN, Hugenholtz P, Tyson GW 2017 Recovery of nearly 8,000 metagenome-assembled genomes substantially expands the tree of life. Nat Microbiol 2:1533–1542. doi:10.1038/s41564-017-0012-7. 28894102

[54] Ortega DR, Zhulin IB 2016 Evolutionary genomics suggests that CheV is an additional adaptor for accommodating specific chemoreceptors within the chemotaxis signaling complex. PLoS Comput Biol 12:e1004723. doi:10.1371/journal.pcbi.1004723. 26844549 PMC4742279

[55] Giovannelli D, Sievert SM, Hügler M, Markert S, Becher D, Schweder T, Vetriani C 2017 Insight into the evolution of microbial metabolism from the deep-branching bacterium, Thermovibrio ammonificans. Elife 6:e18990. doi:10.7554/eLife.18990. 28436819 PMC5441870

[56] Belogurov GA, Sevostyanova A, Svetlov V, Artsimovitch I 2010 Functional regions of the N‐terminal domain of the antiterminator RfaH. Mol Microbiol 76:286–301. doi:10.1111/j.1365-2958.2010.07056.x. 20132437 PMC2871177

[57] Larson MH, Mooney RA, Peters JM, Windgassen T, Nayak D, Gross CA, Block SM, Greenleaf WJ, Landick R, Weissman JS 2014 A pause sequence enriched at translation start sites drives transcription dynamics in vivo. Science 344:1042–1047. doi:10.1126/science.1251871. 24789973 PMC4108260

[58] Shi D, Svetlov D, Abagyan R, Artsimovitch I 2017 Flipping states: a few key residues decide the winning conformation of the only universally conserved transcription factor. Nucleic Acids Res 45:8835–8843. doi:10.1093/nar/gkx523. 28605514 PMC5587751

[59] Beutin L, Manning PA, Achtman M, Willetts N 1981 sfrA and sfrB products of Escherichia coli K-12 are transcriptional control factors. J Bacteriol 145:840–844. doi:10.1128/JB.145.2.840-844.1981. 7007352 PMC217187

[60] Moore D, Wu JH, Kathir P, Hamilton CM, Ippen-Ihler K 1987 Analysis of transfer genes and gene products within the traB-traC region of the Escherichia coli fertility factor, F. J Bacteriol 169:3994–4002. doi:10.1128/jb.169.9.3994-4002.1987. 3040671 PMC213699

[61] Carattoli A 2013 Plasmids and the spread of resistance. Int J Med Microbiol 303:298–304. doi:10.1016/j.ijmm.2013.02.001. 23499304

[62] Sevostyanova A, Belogurov GA, Mooney RA, Landick R, Artsimovitch I 2011 The β subunit gate loop is required for RNA polymerase modification by RfaH and NusG. Mol Cell 43:253–262. doi:10.1016/j.molcel.2011.05.026. 21777814 PMC3142557

[63] Main-Hester KL, Colpitts KM, Thomas GA, Fang FC, Libby SJ 2008 Coordinate regulation of Salmonella pathogenicity island 1 (SPI1) and SPI4 in Salmonella enterica serovar Typhimurium. Infect Immun 76:1024–1035. doi:10.1128/IAI.01224-07. 18160484 PMC2258849

[64] Svetlov D, Shi D, Twentyman J, Nedialkov Y, Rosen DA, Abagyan R, Artsimovitch I 2018 In silico discovery of small molecules that inhibit RfaH recruitment to RNA polymerase. Mol Microbiol 110:128–142. doi:10.1111/mmi.14093. 30069925 PMC6595482

[65] Mayer A, Lidschreiber M, Siebert M, Leike K, Soding J, Cramer P 2010 Uniform transitions of the general RNA polymerase II transcription complex. Nat Struct Mol Biol 17:1272–1278. doi:10.1038/nsmb.1903. 20818391

[66] Svetlov V, Nudler E 2011 Clamping the clamp of RNA polymerase. EMBO J 30:1190–1191. doi:10.1038/emboj.2011.76. 21468097 PMC3094107

[67] Mooney RA, Schweimer K, Rosch P, Gottesman M, Landick R 2009 Two structurally independent domains of E. coli NusG create regulatory plasticity via distinct interactions with RNA polymerase and regulators. J Mol Biol 391:341–358. doi:10.1016/j.jmb.2009.05.078. 19500594 PMC2763281

[68] Tadini L, Jeran N, Peracchio C, Masiero S, Colombo M, Pesaresi P 2020 The plastid transcription machinery and its coordination with the expression of nuclear genome: plastid-encoded polymerase, nuclear-encoded polymerase and the genomes uncoupled 1-mediated retrograde communication. Philos Trans R Soc Lond B Biol Sci 375:20190399. doi:10.1098/rstb.2019.0399. 32362266 PMC7209951

[69] Bailey MJ, Hughes C, Koronakis V 2000 In vitro recruitment of the RfaH regulatory protein into a specialised transcription complex, directed by the nucleic acid ops element. Mol Gen Genet 262:1052–1059. doi:10.1007/pl00008648. 10660066

[70] Gruchota J, Denby Wilkes C, Arnaiz O, Sperling L, Nowak JK 2017 A meiosis-specific Spt5 homolog involved in non-coding transcription. Nucleic Acids Res 45:4722–4732. doi:10.1093/nar/gkw1318. 28053118 PMC5416832

[71] Cavalier-Smith T 2018 Kingdom Chromista and its eight phyla: a new synthesis emphasising periplastid protein targeting, cytoskeletal and periplastid evolution, and ancient divergences. Protoplasma 255:297–357. doi:10.1007/s00709-017-1147-3. 28875267 PMC5756292

[72] Adl SM, Simpson AG, Farmer MA, Andersen RA, Anderson OR, Barta JR, Bowser SS, Brugerolle G, Fensome RA, Fredericq S, James TY, Karpov S, Kugrens P, Krug J, Lane CE, Lewis LA, Lodge J, Lynn DH, Mann DG, McCourt RM, Mendoza L, Moestrup O, Mozley-Standridge SE, Nerad TA, Shearer CA, Smirnov AV, Spiegel FW, Taylor MF 2005 The new higher level classification of eukaryotes with emphasis on the taxonomy of protists. J Eukaryot Microbiol 52:399–451. doi:10.1111/j.1550-7408.2005.00053.x. 16248873

[73] Cavalier-Smith T 1998 A revised six-kingdom system of life. Biol Rev Camb Philos Soc 73:203–266. doi:10.1017/s0006323198005167. 9809012

[74] Kim S, Jung KS, Ryu KH 2006 Automatic orthologous-protein-clustering from multiple complete-genomes by the best reciprocal BLAST hits, p 60–70. *In* Li J, Yang Q, Tan AH (ed), Data mining for biomedical applications. BioDM 2006 Lecture Notes in Computer Science, vol 3916 Springer, Berlin, Germany. doi:10.1007/11691730_7.

[75] Eddy SR 2011 Accelerated profile HMM searches. PLoS Comput Biol 7:e1002195. doi:10.1371/journal.pcbi.1002195. 22039361 PMC3197634

[76] Rambaut A 2012 FigTree v1. 4. Molecular evolution, phylogenetics and epidemiology. http://tree.bio.ed.ac.uk/software/figtree/.

[77] Mendler K, Chen H, Parks DH, Lobb B, Hug LA, Doxey AC 2019 AnnoTree: visualization and exploration of a functionally annotated microbial tree of life. Nucleic Acids Res 47:4442–4448. doi:10.1093/nar/gkz246. 31081040 PMC6511854

[78] Matasci N, Hung LH, Yan Z, Carpenter EJ, Wickett NJ, Mirarab S, Nguyen N, Warnow T, Ayyampalayam S, Barker M, Burleigh JG, Gitzendanner MA, Wafula E, Der JP, dePamphilis CW, Roure B, Philippe H, Ruhfel BR, Miles NW, Graham SW, Mathews S, Surek B, Melkonian M, Soltis DE, Soltis PS, Rothfels C, Pokorny L, Shaw JA, DeGironimo L, Stevenson DW, Villarreal JC, Chen T, Kutchan TM, Rolf M, Baucom RS, Deyholos MK, Samudrala R, Tian Z, Wu X, Sun X, Zhang Y, Wang J, Leebens-Mack J, Wong GK 2014 Data access for the 1,000 Plants (1KP) project. Gigascience 3:17. doi:10.1186/2047-217X-3-17. 25625010 PMC4306014

[79] Soding J, Biegert A, Lupas AN 2005 The HHpred interactive server for protein homology detection and structure prediction. Nucleic Acids Res 33:W244–W248. doi:10.1093/nar/gki408. 15980461 PMC1160169

[80] Katoh K, Rozewicki J, Yamada KD 2019 MAFFT online service: multiple sequence alignment, interactive sequence choice and visualization. Brief Bioinform 20:1160–1166. doi:10.1093/bib/bbx108. 28968734 PMC6781576

[81] Waterhouse AM, Procter JB, Martin DM, Clamp M, Barton GJ 2009 Jalview version 2—a multiple sequence alignment editor and analysis workbench. Bioinformatics 25:1189–1191. doi:10.1093/bioinformatics/btp033. 19151095 PMC2672624

[82] Kumar S, Stecher G, Li M, Knyaz C, Tamura K 2018 MEGA X: Molecular Evolutionary Genetics Analysis across Computing Platforms. Mol Biol Evol 35:1547–1549. doi:10.1093/molbev/msy096. 29722887 PMC5967553

[83] Letunic I, Bork P 2019 Interactive Tree Of Life (iTOL) v4: recent updates and new developments. Nucleic Acids Res 47:W256–W259. doi:10.1093/nar/gkz239. 30931475 PMC6602468

[84] Stamatakis A 2014 RAxML version 8: a tool for phylogenetic analysis and post-analysis of large phylogenies. Bioinformatics 30:1312–1313. doi:10.1093/bioinformatics/btu033. 24451623 PMC3998144

[85] Price MN, Dehal PS, Arkin AP 2009 FastTree: computing large minimum evolution trees with profiles instead of a distance matrix. Mol Biol Evol 26:1641–1650. doi:10.1093/molbev/msp077. 19377059 PMC2693737

[86] Rozewicki J, Li S, Amada KM, Standley DM, Katoh K 2019 MAFFT-DASH: integrated protein sequence and structural alignment. Nucleic Acids Res 47:W5–W10. doi:10.1093/nar/gkz342. 31062021 PMC6602451

[87] Tatusov RL, Koonin EV, Lipman DJ 1997 A genomic perspective on protein families. Science 278:631–637. doi:10.1126/science.278.5338.631. 9381173

[88] Huang Y, Niu B, Gao Y, Fu L, Li W 2010 CD-HIT Suite: a web server for clustering and comparing biological sequences. Bioinformatics 26:680–682. doi:10.1093/bioinformatics/btq003. 20053844 PMC2828112

[89] Katoh K, Standley DM 2013 MAFFT Multiple Sequence Alignment Software Version 7: improvements in performance and usability. Mol Biol Evol 30:772–780. doi:10.1093/molbev/mst010. 23329690 PMC3603318

[90] Camacho C, Coulouris G, Avagyan V, Ma N, Papadopoulos J, Bealer K, Madden TL 2009 BLAST+: architecture and applications. BMC Bioinformatics 10:421. doi:10.1186/1471-2105-10-421. 20003500 PMC2803857

[91] Oliphant TE 2007 Python for scientific computing. Comput Sci Eng 9:10–20. doi:10.1109/MCSE.2007.58.

[92] Team RC 2014 R: a language and environment for statistical computing. R Foundation for Statistical Computing, Vienna, Austria http://www.R-project.org/.

[93] Adebali O, Zhulin IB 2017 Aquerium: a web application for comparative exploration of domain-based protein occurrences on the taxonomically clustered genome tree. Proteins 85:72–77. doi:10.1002/prot.25199. 27802571 PMC5167639

