# Supplementary material for: Origins and Molecular Evolution of the NusG Paralog RfaH
Source: mBio. 2020 Oct 27;11(5):e02717-20. doi: 10.1128/mBio.02717-20 (PMC7593976; doi:10.1128/mBio.02717-20)
Supplement: FIG S1 [file mBio.02717-20-sf001.pdf]

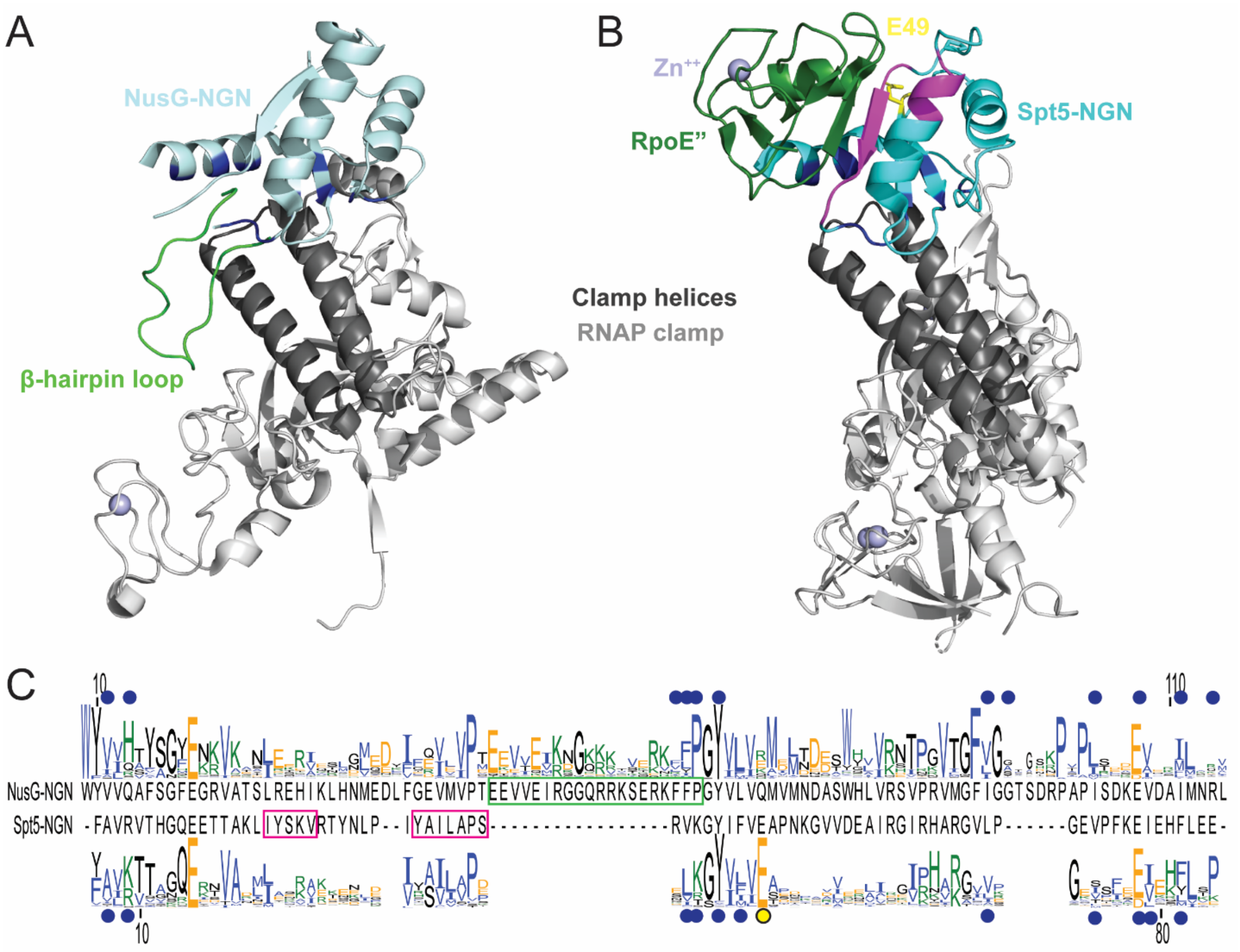

FIG S1 Structural comparison of NusG- and Spt5-NGN. (A) Structure of *E. coli* NusG-RNAP complex (PDB ID: 6C6U). The β’ clamp helices (CH)-binding residues are indicated in blue. Position of the β-hairpin loop was determined by superposition with the NusG structure (PDB ID: 2K06). (B) Structure of *Pyrococcus furiosus* Spt5-NGN-RpoE’’-RNAP complex (PDB ID: 3QQC). RPB1 CH-binding residues are shown in blue. RpoE’’ binding residues - in magenta, with the invariant E49 involved in acid-dipole interaction with Spt4 shown in yellow. (C) Alignment of *E. coli* NusG-NGN (NP_418409.1) and *P. furiosus* Spt5-NGN (WP_011013134.1). NusG (top) and Spt5 (bottom) logos generated from sequences collected from GTDB_reps (sequences were reduced at 90% identity by CD-hit (89); a total of 4,220 NusG and 154 Spt5 sequences were included in the final alignment). CH-binding residues are indicated with blue circles; E49 - with a yellow circle. β hairpin loop and RpoE’’ binding region are shown in green and magenta boxes, respectively.
