# Supplementary material for: Origins and Molecular Evolution of the NusG Paralog RfaH
Source: mBio. 2020 Oct 27;11(5):e02717-20. doi: 10.1128/mBio.02717-20 (PMC7593976; doi:10.1128/mBio.02717-20)
Supplement: FIG S2 [file mBio.02717-20-sf002.pdf]

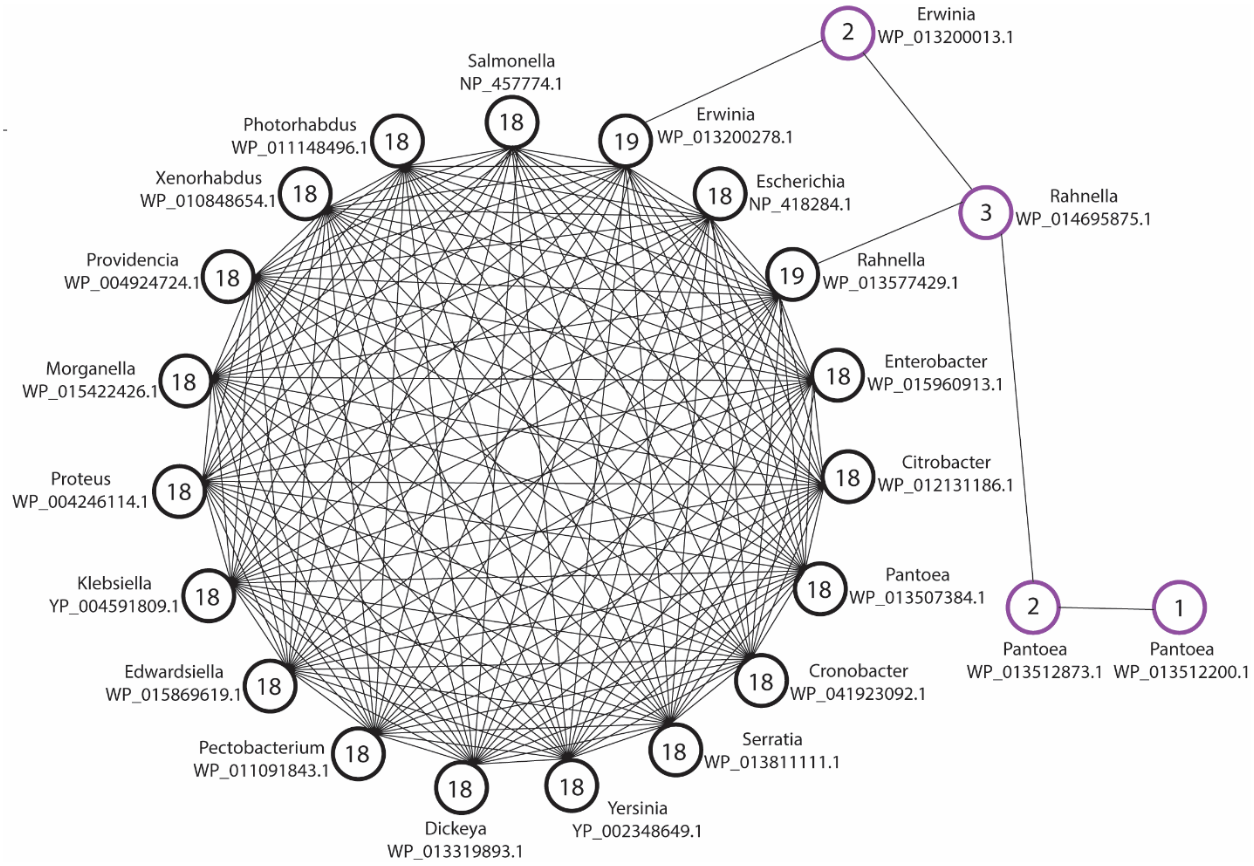

FIG S2 Reciprocal best BLAST hits. Protein sequences were used as query to BLAST against each other's genome. If two sequences find each other as a best scoring match in each other's genome, they represent the reciprocal best BLAST hit and are connected with a line. The number of mutual connections is indicated inside circles. Black circles, chromosomal RfaH. Purple circles, plasmid RfaH. RfaH was presented as genus name and protein NCBI accession number. A high number of observed mutual connections indicates that these representatives belong to the same orthologous groups.
