# Supplementary material for: Origins and Molecular Evolution of the NusG Paralog RfaH
Source: mBio. 2020 Oct 27;11(5):e02717-20. doi: 10.1128/mBio.02717-20 (PMC7593976; doi:10.1128/mBio.02717-20)
Supplement: FIG S3 [file mBio.02717-20-sf003.pdf]

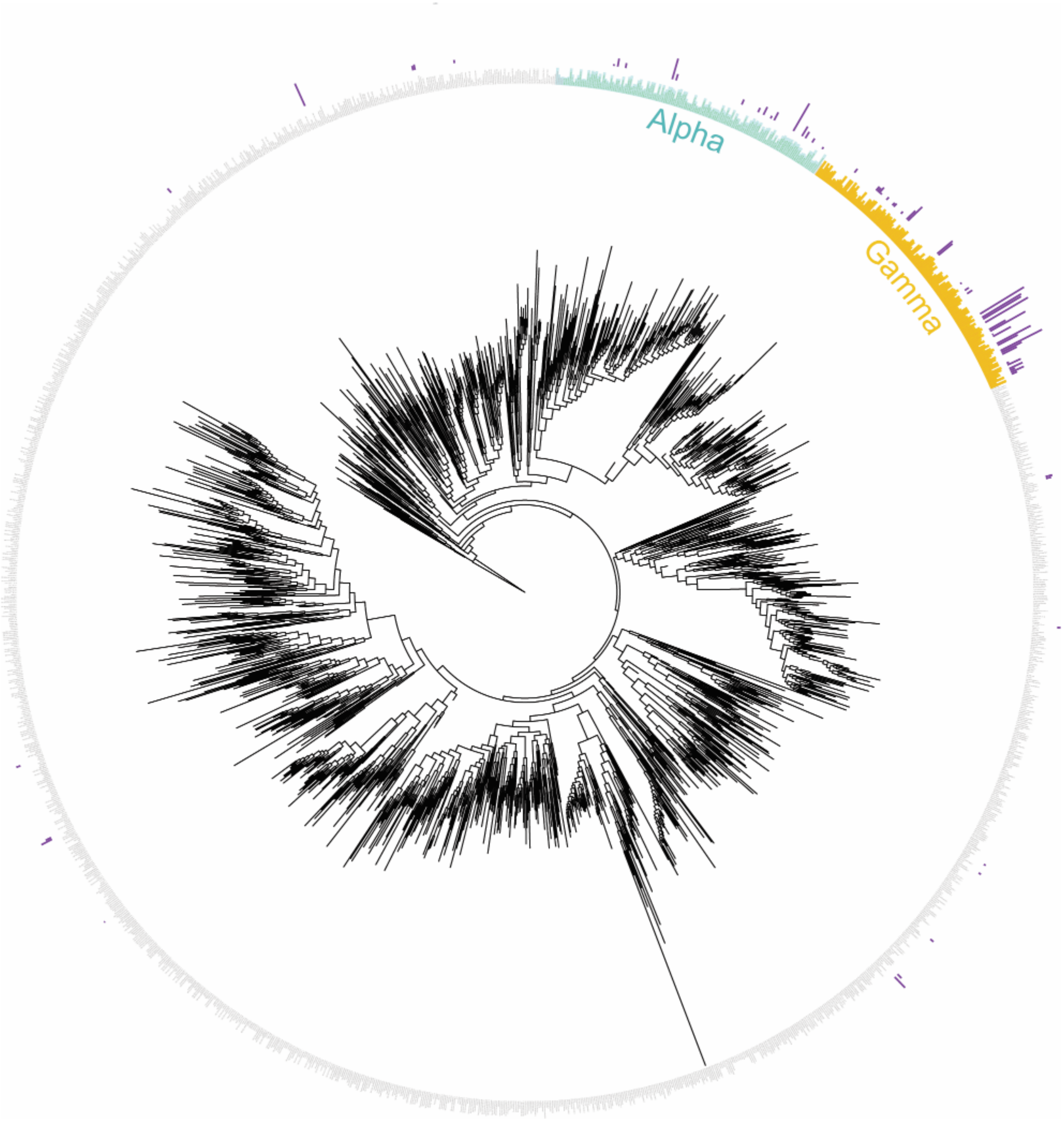

FIG S3 New RfaH model hits on family level of Bacteria. The maximum-likelihood phylogenetic tree was downloaded from AnnoTree (http://annotree.uwaterloo.ca/) (2). The percentage of RfaH hits was calculated for families with ⩾10 genomes and shown as bars on the outer ring. The percentages of RfaH genome hits are high in Gamma- and Alpha-proteobacteria.
