# Supplementary material for: Origins and Molecular Evolution of the NusG Paralog RfaH
Source: mBio. 2020 Oct 27;11(5):e02717-20. doi: 10.1128/mBio.02717-20 (PMC7593976; doi:10.1128/mBio.02717-20)
Supplement: FIG S4 [file mBio.02717-20-sf004.pdf]

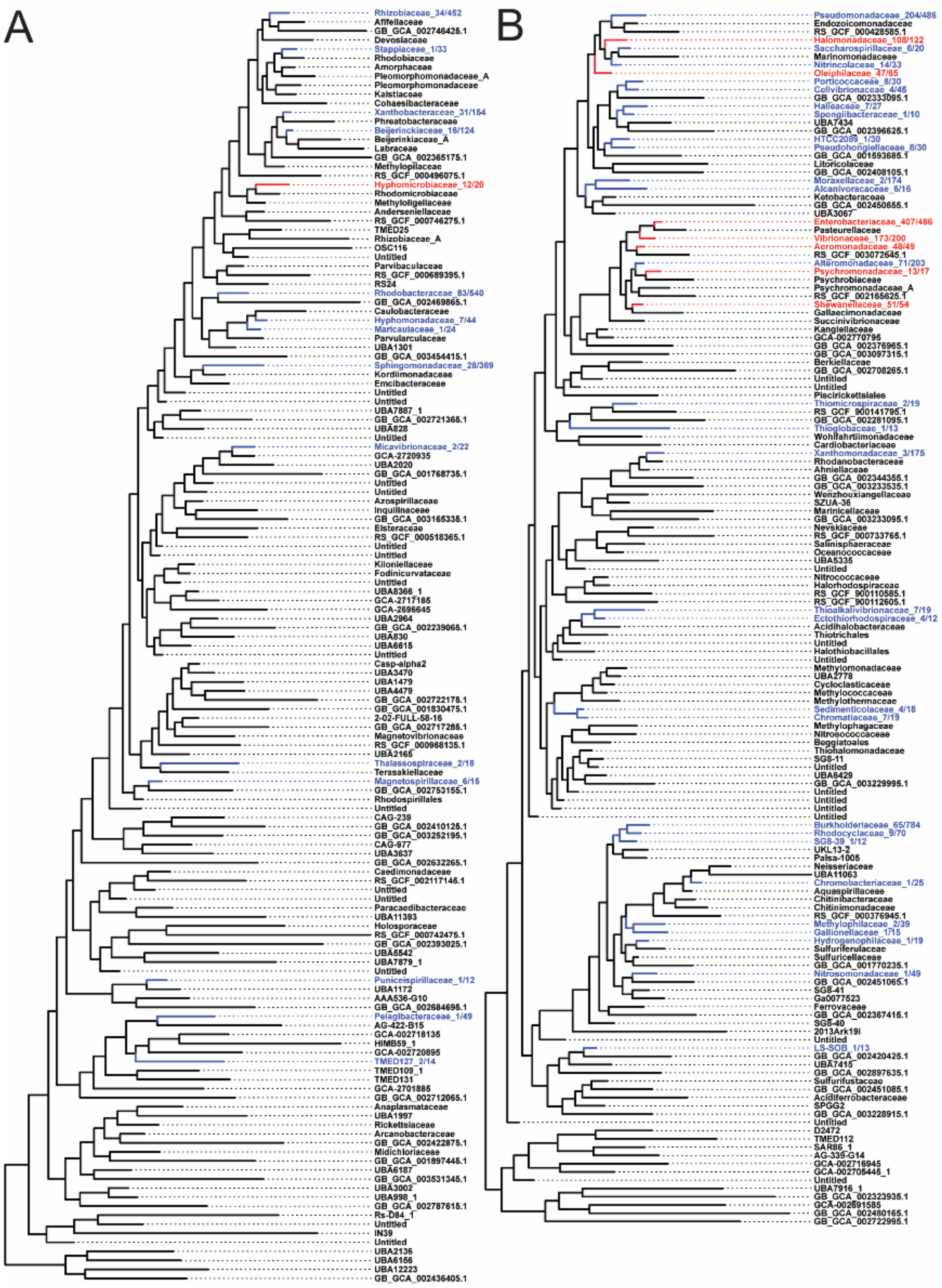

FIG S4 New RfaH model hits in families of Alpha- (A) and Gamma-proteobacteria (B). The maximum-likelihood phylogenetic tree was downloaded from AnnoTree (http://annotree.uwaterloo.ca/) (2). Untitled, no corresponding taxonomy currently. The percentage of genome hits were calculated for RfaH containing families with ⩾10 genomes. Families with > 50% hits are in red, those with < 50% hits in blue.
