# Supplementary material for: Origins and Molecular Evolution of the NusG Paralog RfaH
Source: mBio. 2020 Oct 27;11(5):e02717-20. doi: 10.1128/mBio.02717-20 (PMC7593976; doi:10.1128/mBio.02717-20)
Supplement: FIG S5 [file mBio.02717-20-sf005.pdf]

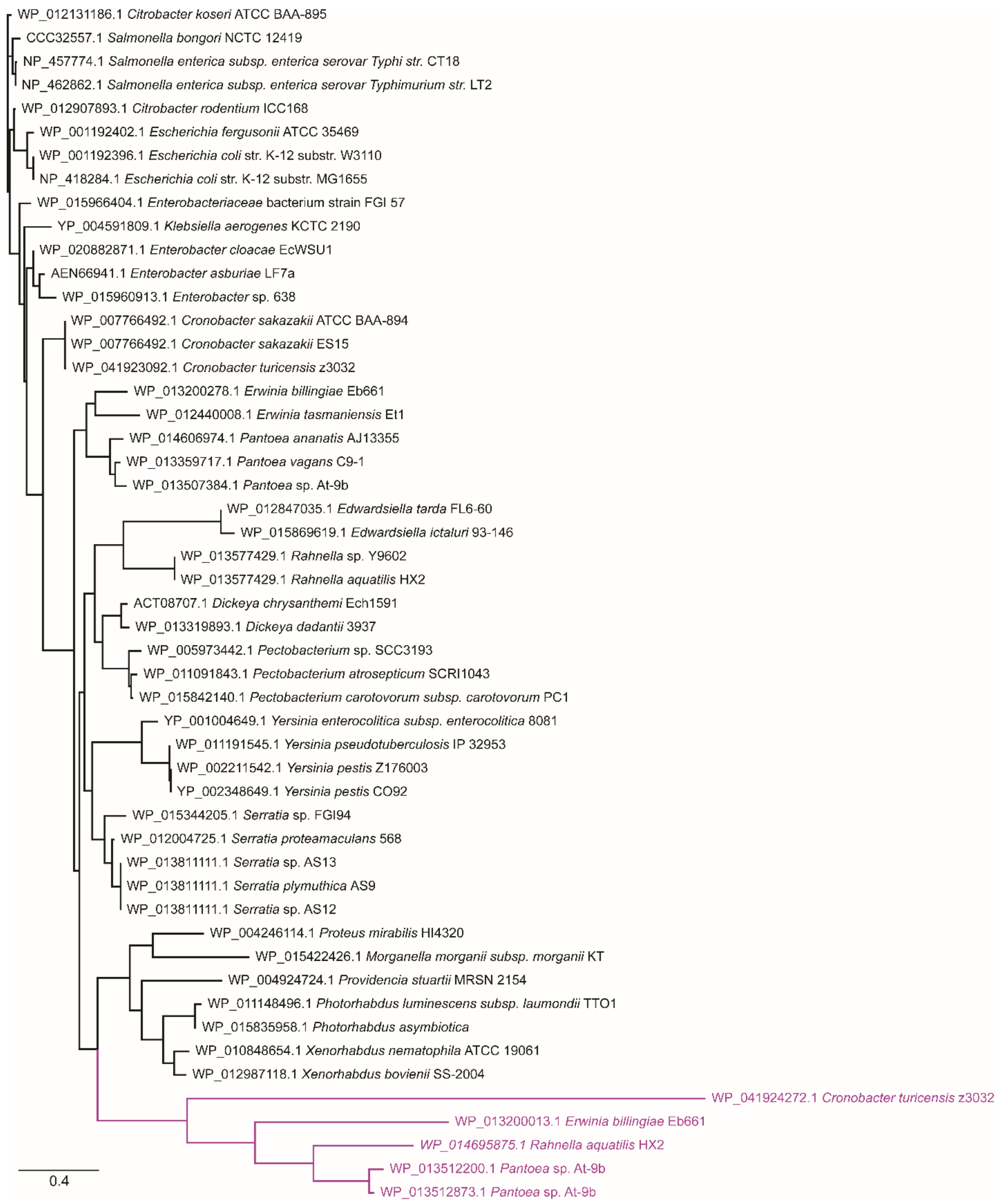

FIG S5 Maximum-likelihood phylogenetic tree of *Enterobacteriaceae* RfaH. The phylogenetic tree was inferred from RfaH sequences by FastTree (86) with JTT model. NCBI accession number of RfaH and organisms' names are shown. The branch of plasmid RfaH is colored in purple. The topology of *Enterobacteriaceae* RfaH tree is similar to the 16S rRNA tree of *Enterobacteriaceae* (Fig. 4).
