# Supplementary material for: Origins and Molecular Evolution of the NusG Paralog RfaH
Source: mBio. 2020 Oct 27;11(5):e02717-20. doi: 10.1128/mBio.02717-20 (PMC7593976; doi:10.1128/mBio.02717-20)
Supplement: FIG S6 [file mBio.02717-20-sf006.pdf]

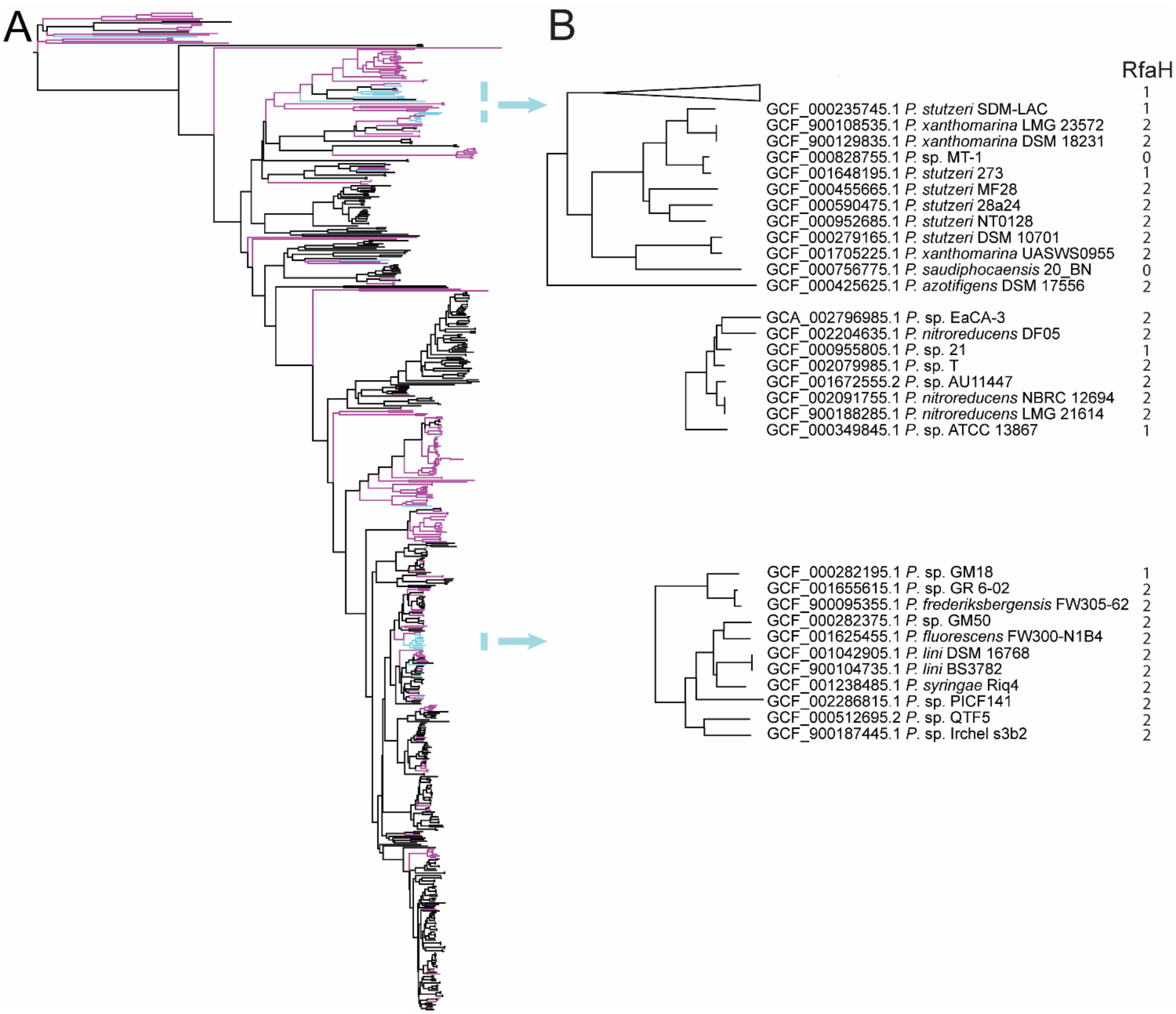


**FIG S6** Distribution of RfaH in *Pseudomonadaceae.* (A) The midpoint rooted maximum-likelihood phylogenetic tree was built based on 120 ubiquitous marker genes for microbial classification (5). The presence of RfaH is highlighted in purple and the ones with RfaH duplication in cyan. (B) Three clades with extensive gene duplications are shown. *P.*, *Pseudomonas*. The number of *rfaH* genes is indicated.
