# Supplementary material for: Origins and Molecular Evolution of the NusG Paralog RfaH
Source: mBio. 2020 Oct 27;11(5):e02717-20. doi: 10.1128/mBio.02717-20 (PMC7593976; doi:10.1128/mBio.02717-20)
Supplement: FIG S7 [file mBio.02717-20-sf007.pdf]

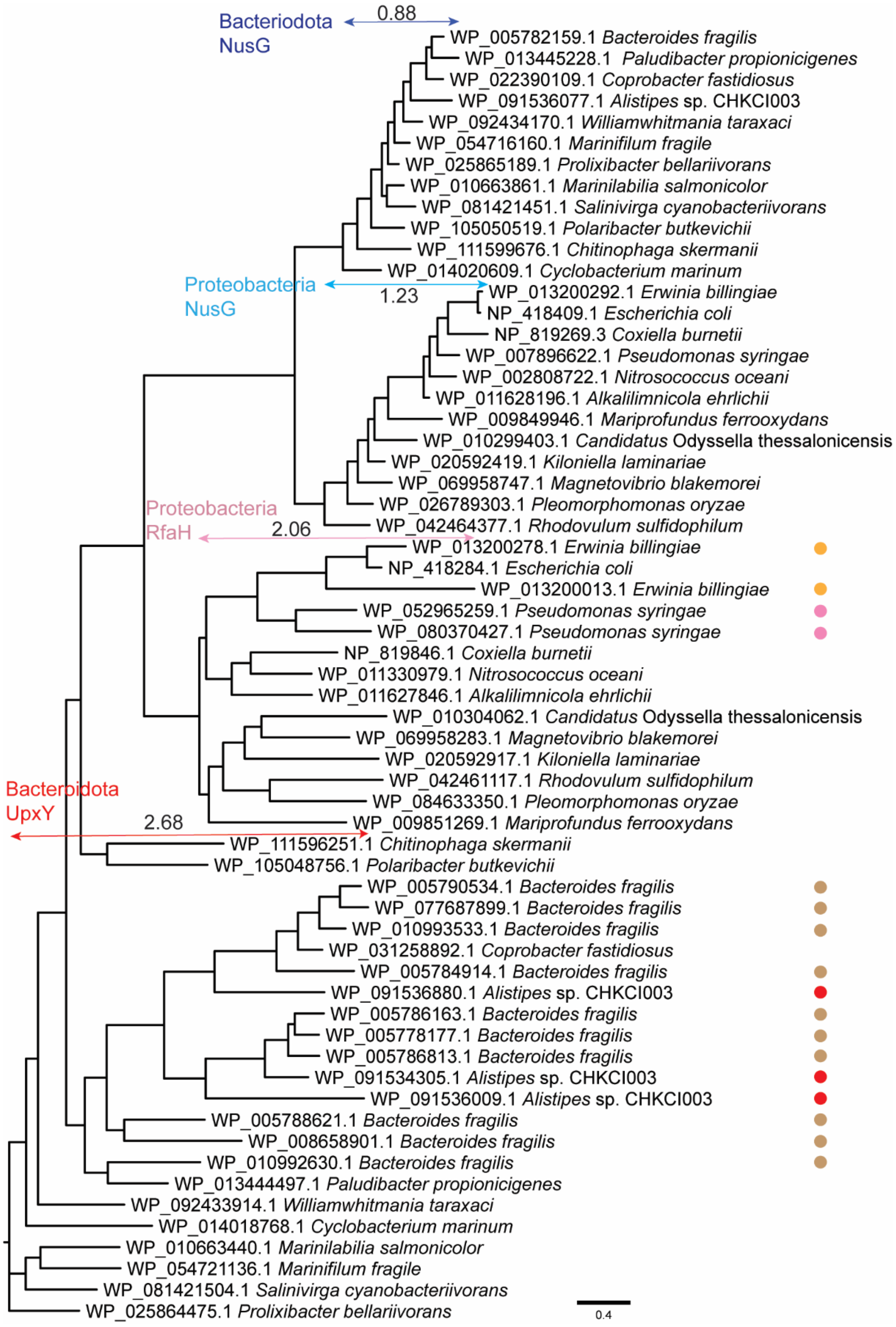

FIG S7 Phylogenetic comparison of NusG, RfaH, and UpxY. The numbers above colored arrows indicate the branch length sum of the longest tree path of NusG homologs. Gene duplications on the same genome are indicated with circles of the same color.
