# Supplementary material for: Origins and Molecular Evolution of the NusG Paralog RfaH
Source: mBio. 2020 Oct 27;11(5):e02717-20. doi: 10.1128/mBio.02717-20 (PMC7593976; doi:10.1128/mBio.02717-20)
Supplement: FIG S8 [file mBio.02717-20-sf008.pdf]

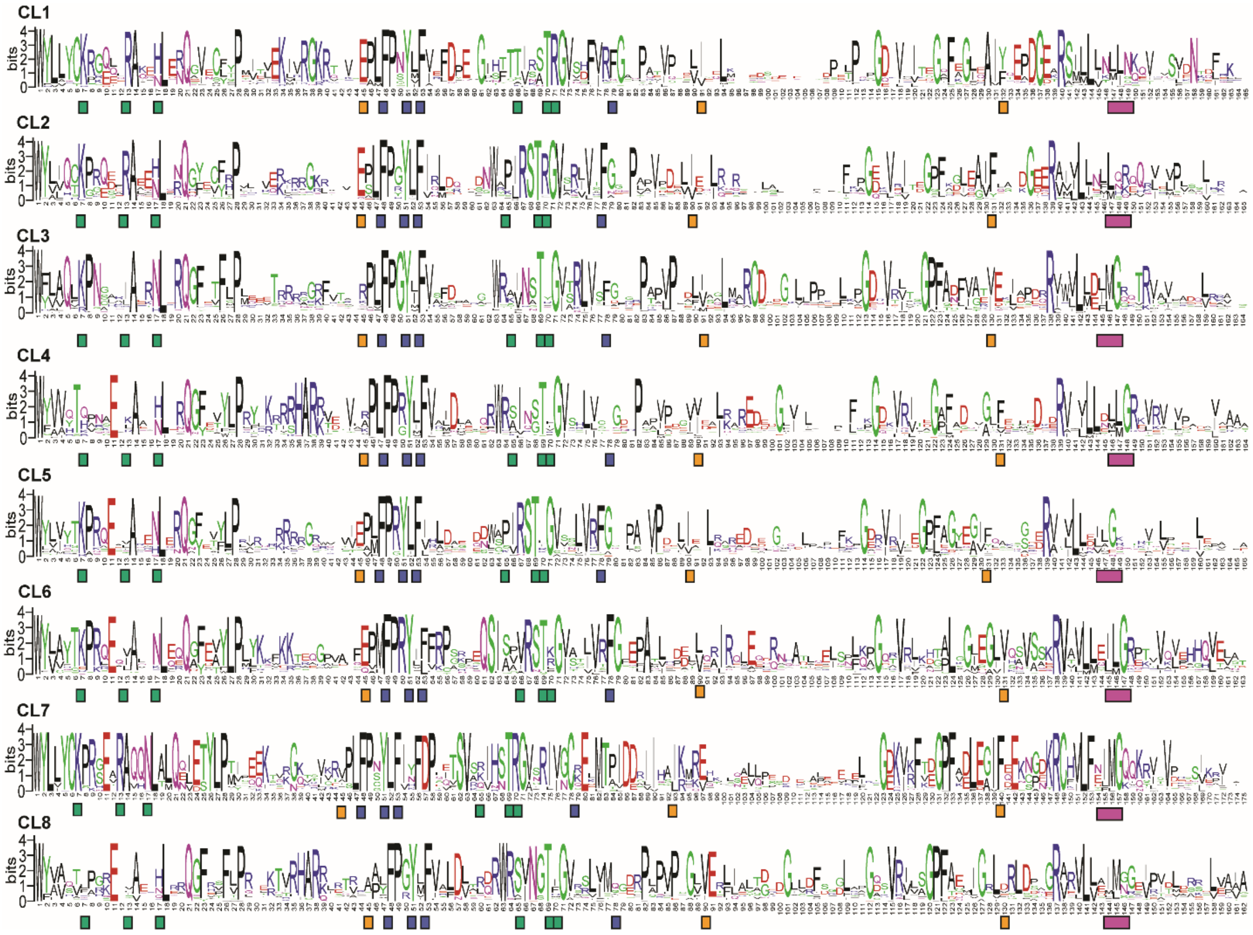

FIG S8 Sequence logos of eight RfaH clusters (CL). The logos were created based on multiple sequence alignments of RfaH sequences from each CL using WebLogo (https://weblogo.berkeley.edu/) (Crooks GE, Hon G, Chandonia JM, Brenner SE, Genome Res 14(6):1188-1190, 2004). The logos were trimmed to begin with the conserved residue tryptophan in all CLs. Functional sites discussed in Fig. 5A are marked with colored squares. Green, *ops* binding sites; Orange, sites responsible for autoinhibition; Blue, RNAP β' CH binding sites; Purple, Rho binding sites.
