# Supplementary material for: Origins and Molecular Evolution of the NusG Paralog RfaH
Source: mBio. 2020 Oct 27;11(5):e02717-20. doi: 10.1128/mBio.02717-20 (PMC7593976; doi:10.1128/mBio.02717-20)
Supplement: FIG S9 [file mBio.02717-20-sf009.pdf]

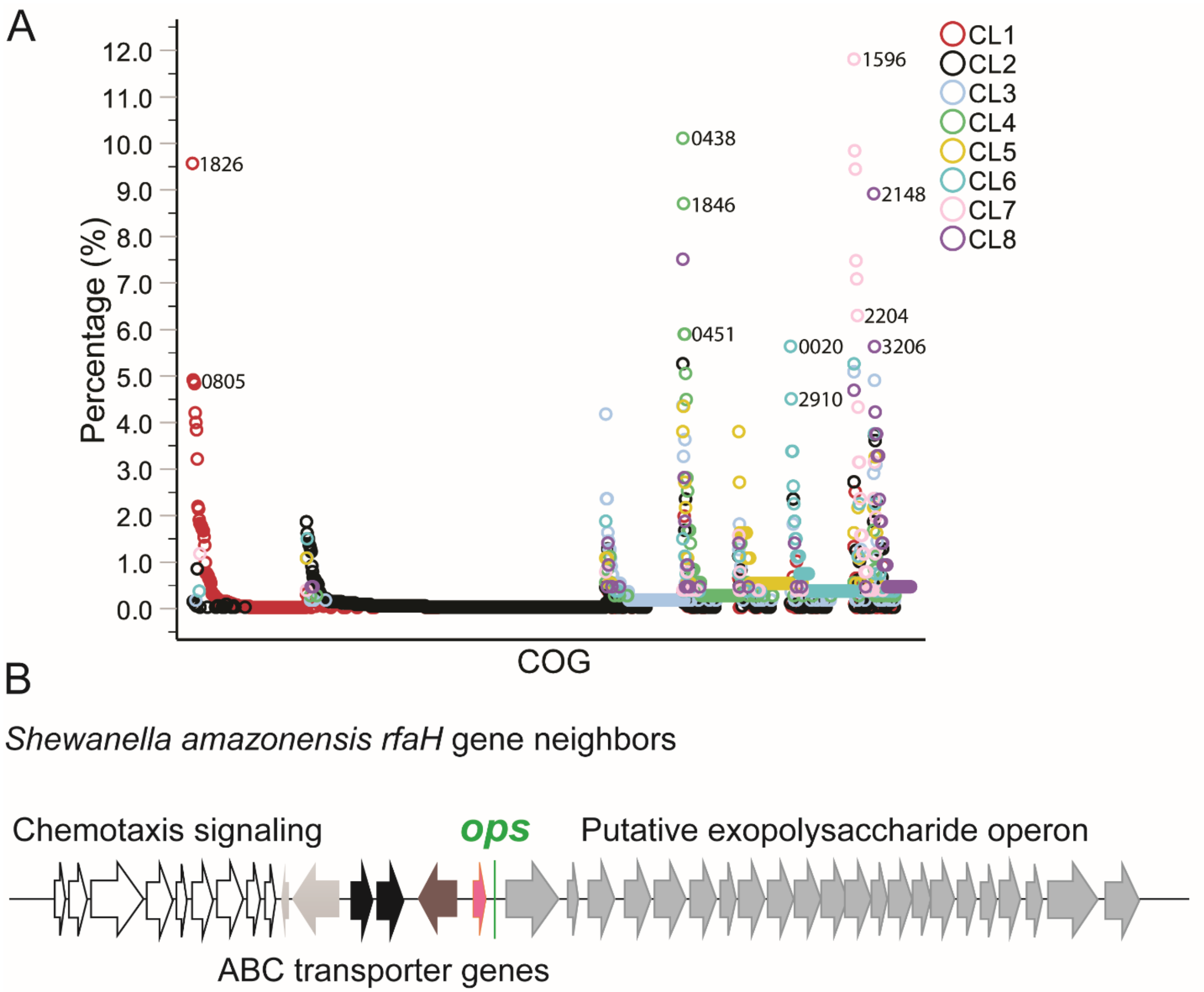

FIG S9 Enrichment of neighboring genes of RfaH clusters (CL). (A) A total of 1,054 COGs were assigned to neighbor genes of *rfaH* (five genes on both sides of *rfaH* genes). Every circle represents a COG. The percentage of COG was calculated as (raw count of COG) / (total neighbor genes of one CL). Then, COGs were assigned with a unique integer in the range of 1 – 1,054 and the same COG in different CL will be assigned with same integer. These integers were used to build X-axis. The identities of highly abundant COGs (41) are indicated. (B) An example of the conversed CL7 *rfaH* location. From left to right: white arrows indicate chemotaxis signaling genes; light brown arrows - type III secretion system-related; black arrows - a transport system; brown arrow - major facilitator superfamily (MFS) transporter; hotpink arrow – *rfaH*; green bar - the *ops* site; and grey arrows - exopolysaccharide operon.
